# Current and Potential Applications of Bismuth-Based Drugs

**DOI:** 10.3390/molecules190915258

**Published:** 2014-09-23

**Authors:** Donal M. Keogan, Darren M. Griffith

**Affiliations:** Centre for Synthesis & Chemical Biology, Department of Pharmaceutical & Medicinal Chemistry, Royal College of Surgeons in Ireland, 123 St. Stephens Green, Dublin 2, Ireland

**Keywords:** bismuth, bismuth complexes, organobismuth compounds, medicinal chemistry, gastrointestinal, *H. pylori*, anti-microbial, anti-leishmanial, anti-cancer

## Abstract

Bismuth compounds have been used extensively as medicines and in particular for the treatment of gastrointestinal ailments. In addition to bismuth’s well known gastroprotective effects and efficacy in treating *H. pylori* infection it also has broad anti-microbial, anti-leishmanial and anti-cancer properties. Aspects of the biological chemistry of bismuth are discussed and biomolecular targets associated with bismuth treatment are highlighted. This review strives to provide the reader with an up to date account of bismuth-based drugs currently used to treat patients and discuss potential medicinal applications of bismuth drugs with reference to recent developments in the literature. Ultimately this review aims to encourage original contributions to this exciting and important field.

## 1. Introduction

**Figure 1:**
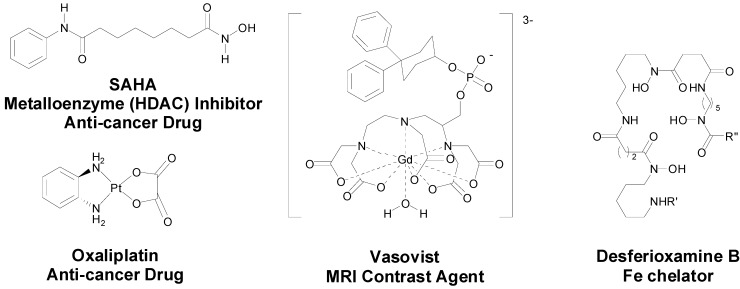
Structures of representative examples of a metal-based therapeutic and diagnostic, metal chelator and metalloprotein inhibitor [3,6].

The use of metal-based drugs can be traced back to ancient times and has a rich and varied history [1]. Gold-based medicines were being used in China and the Middle East as far back as 3500 years ago and mercurous chloride (Hg_2_Cl_2_) was used as a diuretic during the Renaissance period, for example [2,3]. More recently in the early twentieth century Paul Ehrlich, who coined the term “chemotherapy”, developed Salvarsan, an arsenical, as a drug for the treatment of syphilis [4]. Contemporary medicinal inorganic chemistry though is a relatively young but vibrant research discipline. There are many excellent examples of metal-based therapeutics and diagnostics, metal chelators and inhibitors of metalloproteins in the clinic, for example Figure 1, or in development and our knowledge of the roles of metal ions in biological systems is continuing to expand [3,5].

The rational design of metal-based drugs is desirable. Careful selection of metals and manipulation of geometries, coordination numbers and redox states by selection of appropriate ligands can lead to the regulation of electronic, chemical and photophysical properties. Individual ligands also contribute greatly to structural diversity and modulate stability, ligand exchange kinetics and second coordination sphere interactions. In turn the prediction and control of the pharmacodynamics and pharmacokinetics of compounds together with the developments in relation to metallomics provide the opportunity to develop metal-based drugs with bespoke mechanisms of action [3,5].

Bismuth (Bi) has no known natural biological role, though there are references to Bi-containing medicines dating back to the late 18th century [7], and by the 20th century numerous different bismuth preparations were used as treatments for a variety of disorders including the treatment of war wounds, cholera infantum and gastroenteritis [8,9,10]. One of these preparations is well known and still in use today. Pepto-Bismol (bismuth subsalicylate, BSS), developed in 1901, is currently used to treat gastritis and dyspepsia. Other current bismuth medications include De-Nol (colloidal bismuth subcitrate, CBS) and the more recently developed Pylorid (ranitidine bismuth citrate, RBC) which are used for the treatment of ulcers and *H. pylori* infection [3].

Bismuth, thought to derive from the German word “wismuth” (white mass) is a relatively rare element and is the least abundant of the pnictogens or group 15 elements. In 1753 Claude Francois Geoffroy demonstrated bismuth to be a distinct element, as opposed to being elemental tin or lead. It is primarily found as bismuthinite (bismuth sulfide) and bismite (bismuth oxide) ores and sourced as a by-product of lead, copper and tin mining. It is naturally monoisotopic (^209^Bi) and can be considered to be the heaviest stable element, given its theoretical half-life is 1.9 × 10^9^ years [11]. Radioactive bismuth-212 (^212^Bi) and bismuth-213 (^213^Bi) in contrast have half-lives of 60.6 and 45.6 min respectively, and may have important roles to play as radiometals in radiopharmaceuticals [3,12].

The metallic character of the group 15 elements increases down the group and Bi is considered metallic (or semi-metallic), as opposed to arsenic and antimony which are considered to be metalloids and nitrogen and phosphorous to be non-metals. The chemistry of arsenic, antimony and Bi is certainly less understood than that of nitrogen and phosphorous. Bi has a ground state electronic configuration of [Xe]4f^14^5d^10^6s^2^6p^3^ and trivalent and pentavalent Bi, Bi(III) and Bi(V), respectively, are the two predominant oxidation states. In general the three 6p electrons are involved in bond formation and in the majority of compounds Bi is in the +3 oxidation state. Such compounds possess a so-called inert 6s^2^ pair of electrons, which can have a stereochemical effect. These two 6s electrons can be involved in bonding though and many examples of organobismuth(V) compounds are known [13]. BiF_5_ is the only Bi(V) halide known. Given Bi(V) is a very strong oxidant in aqueous solutions (Bi^V^/Bi^III^ E^0^ = 2.03 V) it is noteworthy that Bi(V) is generally not stable in biological solutions [14].

Organobismuth compounds have at least one direct carbon to Bi bond and represent an important class of organometallic compounds. The chemistry of organobismuth(III) and (V) compounds is continuing to develop and many classes have been reported including bismuth(III) containing-heterocylces (bismacycles and heterobismacycles) and triphenylbismuth(V) bis(carboxylate) complexes for instance [15,16,17].

Two key factors contribute to the coordination chemistry of Bi(III): (i) the 6s orbital is stabilized by relativistic effects resulting in the two 6s^2^ electrons being less readily available for bonding and a concomitant reduction in Lewis basicity (inert pair effect) and (ii) the coordination around Bi(III) centres, particularly when Bi(III) is bonded to electronegative atoms or groups, can be extended due to the significant degree of Lewis acidity as a result of: (a) the availability of unoccupied d and/or Bi-X σ* orbitals (where X is a halide for example) and (b) weak shielding of the 4f electrons, as per the lanthanide contraction [13,14]. Bi(III) is classified as a borderline Lewis acid according to the Hard Soft Acids Bases (HSAB) theory and therefore can form weak complexes with hard Lewis bases for example [18]. In general though Bi(III) is known to have a high affinity for oxygen, nitrogen and sulphur ligands. An affinity for thiolate ligands is considered to be an important property of Bi(III) in biological systems [14]. Bi(III) also has a relatively large ionic radius (1.16 Å), which can facilitate the formation of interesting complexes with higher coordination numbers and a wide range of geometries [19,20].

In regards to the biological chemistry of Bi the identification of potential targets of Bi-based drugs should be more readily achievable given the developments in relation to metallomics. It is well-established though that Bi can interact with: (i) nucleosides/nucleotides; (ii) amino acids/peptides and in turn (iii) proteins/enzymes [14].

**Figure 2:**
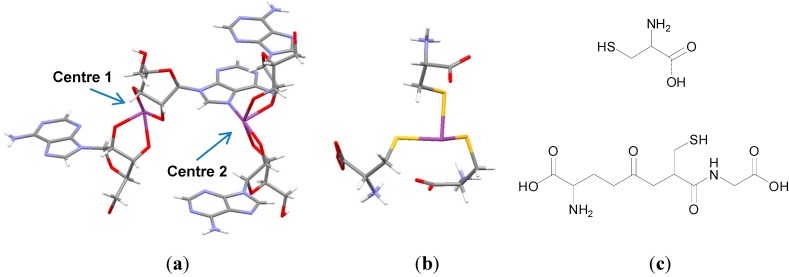
(**a**) Crystal structure of a Bi adenosine complex (CCDC: 634530) [21]; (**b**) Crystal Structure of Bi(Cys)_3_ (CCDC: 685969) [23]; Colour code:  C, grey; H, white; O, red; N, blue; S, yellow; Bi, purple; (**c**) Structures of cysteine (top) and glutathione (bottom).

Bismuth, for example, has been demonstrated to complex nucleosides as elegantly demonstrated by the crystal structure solved in Figure 2a. One Bi(III) centre (centre 1) is shown to bind to two adenosines via the deprotonated *cis*-2',3'diol groups and a second centre (centre 2) binds two adenosines via the deprotonated *cis*-2',3'diol groups and the nucleobase adenine via the imidazole N [21].

In addition a bismuth(III) complex of 1,4,7,10-tetrakis(2-pyridylmethyl)-1,4,7,10-tetraazacyclododecane (TPC) was demonstrated to bind calf thymus DNA. This interaction though is likely to be non-covalent interchelative [22].

Bi(III) as mentioned has an affinity for thiolate ligands and thiolation of Bi(III) is thought to be the major biochemical fate of Bi(III) in cells and biological fluids [14,24]. Bi(III) readily binds to the cysteine thiolate groups of amino acids and peptides for example. The crystal structure of Bi(Cys)_3_ was solved by X-ray crystallography demonstrating that Bi(III) can bind three cysteine thiolate groups and at physiological pH (Figure 2b). The average Bi-S bond length is *ca.* 2.54 Å [23]. Furthermore Bi(III) has been demonstrated to bind to glutathione (GSH), a tripeptide antioxidant, which is found at high concentrations in animal cells (*ca.* 5 mM). ESI-MS data indicates that Bi(III) and GSH bind at ratios of 1:1, 1:2 and 1:3 [25]. A previous study by Sadler *et al.* had shown that the deprotonated thiol groups of GSH were the strongest binding site for Bi(III) and Bi(GSH)_3_ is stable over a pH range of 2–10. A formation constant for Bi(GSH)_3_ of log K = 29.6 was determined, using ^1^H-NMR upon titration of GSH with [Bi(Hedta)]. Bi(GSH)_3_ is therefore more stable than Bi(III) complexes with edta (log K = 27.8) or citrate (log K = 13.5) for example [24]. Significantly spin-echo ^1^H-NMR experiments demonstrated that RBC reacts with GSH in red blood cells both *in vivo* and *in vitro* [24]. There are numerous studies of Bi(III) binding to longer peptide sequences [26,27,28].

**Figure 3:**
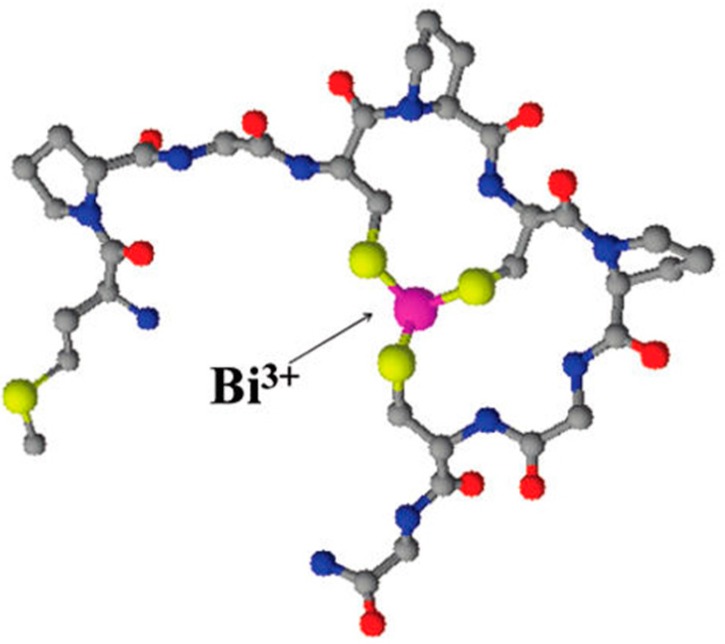
Proposed structures of BiL complex; L is the N-terminus of ZIP13 (MPGCPCPGCG-NH_2_). Hydrogens were removed for clarity; colours: grey, carbon; blue, nitrogen; red, oxygen; yellow, sulphur [29]. Reproduced with permission from [29]. Copyright (2011) American Chemical Society.

Potocoki and co-workers investigated the coordination modes and thermodynamic stabilities of Zn(II), Cd(II), Bi(III), and Ni(II) complexes of the cysteine-rich N-terminal domain fragment of the ZIP13 zinc transporter (MPGCPCPGCG-NH_2_) by potentiometry, mass spectrometry, NMR, CD, and UV-Vis spectroscopy [29]. Bi(III) had the strongest affinity for the peptide sequence studied and the three cysteine thiolate groups as expected were identified as playing key roles in binding, Figure 3 [29].

Considerable research has been undertaken in relation to Bi(III) interactions with proteins and enzymes such as human serum transferrin (hTF) [30,31,32,33,34], metallothionein (MT), [31,35,36]. SARS coronavirus helicase [31,35,36], histidine rich protein (Hpn) [37,38], heat shock protein A (HspA) and urease [39,40,41] amongst others.

**Figure 4:**
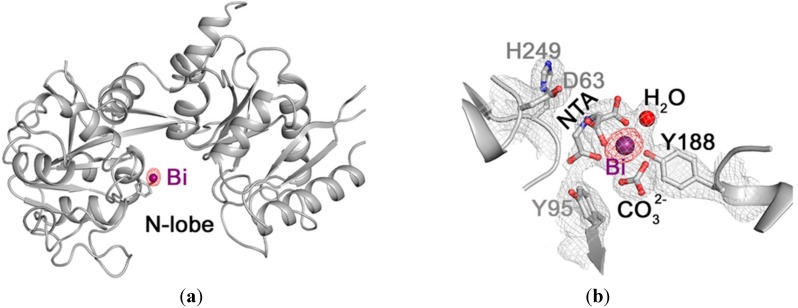
(**a**) Anomalous electron density of Bi_N_Fe_C_-hTF. The hTF backbone is shown in grey ribbon with the residue Tyr188 and Bi(III) represented as stick and sphere models respectively. The anomalous electron density map (contoured at 10 σ), calculated from diffraction data collected at 0.92000 Å, is shown as red mesh and indicates the location of atoms that strongly absorb X-ray photons of this energy; (**b**) Coordination of Bi(III) in the N-lobe of Bi_N_Fe_C_-hTF. The gray 2*F*_obs_ − *F*_calc_ map is contoured at 1.0 σ and the red anomalous electron map is contoured at 10.0 σ. The side chains of putative binding residues Asp63, Tyr95 and His249 are 6.7, 9.9 and 5.5 Å away from the Bi(III), respectively. Reproduced with permission from [42]. Copyright (2012) Nature Publishing Group.

hTF is an iron-transport glycoprotein. It binds Fe(III) tightly but reversibly and plays a key role in iron homeostasis, transporting Fe(III) from extracellular fluid to cytosol [42]. It is found at high concentrations in blood plasma and given it is generally only *ca*. 30% saturated with Fe(III) it can bind other metals such as Bi(III), Ru(III), Cu(II), Ni(II) and Zn(II) [14]. Yang *et al.* recently reported the crystal structure of a Bi-bound hTF (Bi_N_Fe_C_-hTF) where the Bi(III) is found at the N-lobe, as per Figure 4a, and the Fe(III) at the C-lobe. The Bi(III) centre is bound by one tyrosine residue (Tyr188), a bidentate carbonate anion (synergistic anion), a tridentate nitrilotriacetate (NTA_-3H_) and a water molecule, Figure 4b. Bi(NTA_-3H_) was used in the preparation of Bi_N_Fe_C_-hTF [42]. Significantly in addition to providing valuable information on the binding mode of Bi(III) with hTF, this structure also strongly supports the theory that hTF does have a potential role in Bi-based drug delivery.

Given the historical and contemporary contributions of Bi to medicinal chemistry, together with its interesting biological chemistry and reputation as a “green” non-toxic metal [11], there has been much research undertaken to date in relation to the development of novel Bi-based drugs as gastrointestinal, anti-microbial, anti-leishmanial and anti-cancer agents. There also has been a number of excellent reviews and books published in relation to the chemistry [13], biological chemistry [14] and medicinal properties of Bi-based compounds [43,44,45,46]. Though far from an exhaustive account this review strives to provide the reader with an up to date insight into the state of the art of Bi compounds with current and potential medicinal applications and encourage contributions to this important field.

## 2. Gastrointestinal

While bismuth medications were widely used at the start of the 20th century, for example for the treatment of syphilis, demand declined dramatically after the development of antibiotics. This decline continued until the discovery of *H. pylori* in the 1980s after which bismuth emerged as a component of effective treatments for many gastrointestinal disorders [47]. The efficacy of the bismuth preparations in the treatment of many of these disorders has been attributed to a number of different actions against *H. pylori* and cytoprotective effects in the gastric mucosa.

*H. pylori* is a microaerophilic and neutralophilic Gram negative bacterium that has the ability to colonise the human stomach. It was first identified as a potential pathogen in 1984 when it was linked with idiopathic gastritis, inflammation of the lining of the stomach, by Marshall and Warren [47]. Subsequently *H. pylori* has become associated with dyspepsia, peptic ulcer and gastric cancer [48]. More recently *H. pylori* has been linked with extragastric diseases such as cardiovascular diseases, diabetes mellitus, sideropenic anemia, idiopathic thrombocytopenic purpura (ITP), autoimmune diseases and gallbladder cancer for example [49]. It is estimated that about 50 percent of the world’s population is infected with *H. pylori*, though the majority of infected people (>80%) do not develop any associated diseases [50].

*H. pylori* has developed a number of ways to survive and colonise the harsh acidic environment of the gastric mucosa and in turn induce chronic infection. One of these mechanisms is “acid acclimation” whereby in the acidic environment of the stomach, periplasmic pH is adjusted by regulation of urease, UreI (a pH-gated urea channel in the inner membrane of the gastric surface), and α-carbonic anhydrase [51].

First-line treatment for *H. pylori* is a standard triple therapy drug treatment: two antibiotics such as clarithromycin, amoxicillin, tetracycline or metronidazole in addition to a proton pump inhibitor (PPI) [52]. Significantly *H. pylori* infections are becoming increasingly difficult to eradicate due to drug resistance associated with overuse of antibiotics or non-adherence to treatment regimens [53,54]. Bi-containing quadruple therapies therefore are being increasingly recommended as the first-line treatment in a number of countries [55]. As previously mentioned clinically used medications include bismuth subsalicylate (Pepto Bismol, BSS) colloidal bismuth subcitrate (De-Nol, CBS) and the more recently developed ranitidine bismuth citrate (Pylorid, RBS). Many of these Bi-based medicines are administered however without an exact understanding of their structure, behaviour in biological environments or indeed their mechanisms of action.

**Figure 5:**
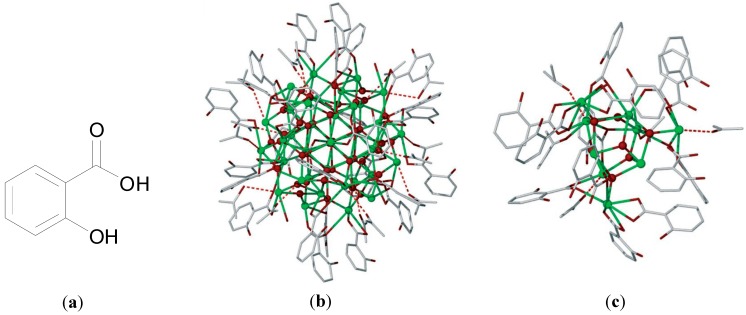
(**a**) Structure of salicylic acid; (**b**) Molecular structure of [Bi_38_O_44_(HSal)_26_(Me_2_CO)_16_(H_2_O)_2_] showing the shrouding of the Bi_38_O_44_ core (green = Bi, red = O) by 26 salicylate ligands (stick structures); (**c**) Molecular structure of [Bi_9_O_7_(HSal)_13_(Me_2_CO)_5_] (**2**) showing the shrouding of the Bi_9_O_7_ core (green = Bi, red = O) by 13 salicylate ligands (stick structures) [56]. Subfigures **b** and **c** are reproduced from [56] with permission from John Wiley and Sons, 2006.

Despite being in medicinal use for over 100 years the exact structure of BSS is not fully understood. In 2006 Andrews and co-workers though reported an important X-ray structure, which provides an insight into the possible chemical nature of BSS. The structures were produced upon crystallisation of [BiHSal_3_], in acetone where H_2_Sal is salicylic acid, Figure 5a. The structures feature BiO clusters of formula [Bi_38_O_44_(HSal)_26_(Me_2_CO)_16_(H_2_O)_2_], Figure 5b, and [Bi_9_O_7_(HSal)_13_(Me_2_CO)_5_], Figure 5c. Each of these clusters contains a BiO core ranging from Bi_9_O_7_ to Bi_38_O_44_ for example [56]. 

**Figure 6:**
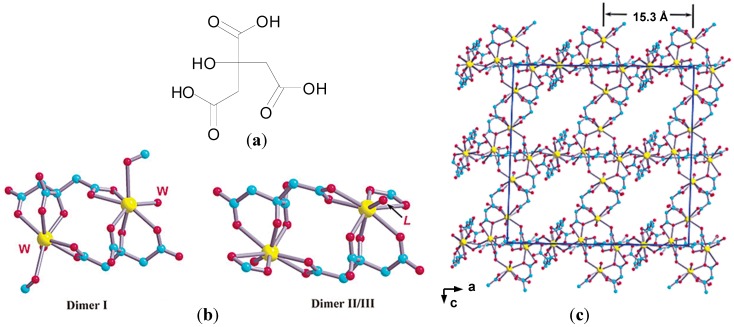
(**a**) Structure of citric acid; (**b**) Basic dimeric units [Bi(cit)_2_Bi]^2−^ found in K(NH_4_)[Bi_2_(cit)_2_(H_2_O)_2_].4H_2_O with a C2 symmetry (dimer I) and an inversion center (dimer II/III). In dimer I, “W” represents a water molecule, while in dimer II/III, the “L” represents either an oxygen from a water molecule (dimer II) or a citrate carboxylate (dimer III). Colour code:  C, cyan; O, red; Bi, yellow; (**c**) Projection of the X-ray structure of K(NH_4_)[Bi_2_(cit)_2_(H_2_O)_2_].4H_2_O down the b axis. All potassium and ammonium ions, hydrogen atoms, and free lattice water molecules are omitted for clarity [62]. Subfigures **b** and **c** are reprinted with permission from [62]. Copyright (2003) American Chemical Society.

Similarly CBS also presents challenges in regards to its structural characterization. Many different bismuth citrate complexes have been isolated and characterized by X-ray crystallography [57,58,59,60,61,62]. Given that speciation of Bi(III) citrate complexes is pH dependent, Jin *et al.* solved the structure of CBS in dilute HCl (pH = 3) using X-ray crystallography. Crystals of K(NH_4_)[Bi_2_(cit)_2_(H_2_O)_2_].4H_2_O revealed the presence of three types of Bi dinuclear units [Bi(cit)_2_Bi]^2−^, which assemble into a 3-D polymer, Figure 6b,c [62]. It was proposed that CBS may rearrange from colloidal particles such as [Bi_6_O_4_(cit)_4_]^6−^ and [Bi_12_O_8_(cit)_8_]^12−^ at neutral pH to 3-D polymers and sheets at low pH and the polymeric structure described may represent the protective “coating” found on ulcer craters post treatment [62]. The channels associated with the polymer matrix may also accommodate molecules of the histamine H_2_-receptor antagonist, ranitidine, in the widely used RBC.

The bismuth carboxylate compounds, for example BSS, CBS, or RBC hydrolyse in the stomach forming insoluble bismuth salts or polymers, which are free to exert their bactericidal effects. BSS for example hydrolyses to release salicylic acid with the concomitant formation of bismuth oxychloride in gastric juice at pH 3.

A single mechanism of action for the bactericidal activity in relation to *H. pylori* has yet to be established although experimental evidence suggests inhibition of the enzymatic activity of urease may be important [40,41,63]. Urease is a dinuclear nickel(II) enzyme, which as discussed previously plays a key role in acid-acclimation, facilitating the bacteria to grow in the highly acidic stomach. Zhang *et al.* for instance demonstrated both competitive and non-competitive inhibition of urease by Bi(III) from a number of complexes including RBC [41]. Using NMR spectroscopy and site-directed mutagenesis studies they suggest that bismuth(III) can bind to the highly conserved cysteine residue (Cys_319_) located at the entrance of the urease active site [41].

Bi has also been shown to have a high affinity for two proteins associated with nickel homeostasis; Heat Shock Protein A (HspA) and *H. pylori* nickel binding protein (Hpn) [40,63,64]. Such interactions could also have knock-on effects on urease (a dinuclear Ni(II) enzyme) activity. [65,66,67,68]. HspA for instance is a molecular chaperone and assists with protein folding processes within the bacterial cell. It was also proposed to be involved in nickel homeostasis in *H. pylori* due to the presence of a nickel binding domain, a His and Cys rich C terminus, and the dependence of urease activation on the HspA gene. Bi(III) has also been reported to binds to apo-HspA with higher affinity than nickel(II) and induce structural reorganisation of the protein [64]. More recently it was proposed that irreversible binding of Bi(III) to an apical domain on HspA consisting of His_45_, Cys_51_ and Cys_53_ residues could interfere with zinc(II) ion delivery functions associated with HspA [69].

Additionally, CBS has also been shown to inhibit alcohol dehydrogenase (ADH) in *H.* *pylori*. ADH can produce acetaldehyde which forms adducts with phospholipids and exogenous proteins thereby damaging gastric cells and causing mucosal damage. Jin *et al.* suggest that inhibition of ADH is due to direct interference at the zinc binding sites through interaction with thiol groups [65,66,67,68]. Bi is also known to interact with/inhibit fumarase [70] translational factor Ef-Tu [71] phospholipase A [72] and pepsin [73]. Though the exact mechanism of action of the anti-bacterial activity associated with Bi has yet to be elucidated, the majority of available evidence points towards Bi interacting with cysteine residues on key proteins or Bi interference at thiol-containing metal binding sites in various target proteins in *H. pylori*.

The precise mechanism by which Bi-based compounds heal ulcers is also not understood. Wagstaff proposed that CBS facilitates healing of the lesion by: (i) forming a Bi-glycoprotein complex *in vitro*, which acts as a protective coating and a barrier to HCl diffusion in the ulcer crater; (ii) stimulating prostaglandin E2 production with consequent secretion of alkali into the mucus layer; (iii) exhibiting an anti-bacterial effect against *H. pylori* as previously discussed [74]. The anti-ulcer effects of CBS may well be more complicated than straightforward precipitation at the ulcer crater though given it is known that even though CBS forms an insoluble precipitate at acidic pH, Bi is taken up by gastric and intestinal mucosal tissue and Bi is capable of interacting with numerous cellular proteins [14]. Further studies are undoubtedly required.

Andrews and co-workers, subsequent to providing an invaluable insight into the possible structure of BSS [56], developed numerous families of novel Bi complexes with excellent anti-*H. pylori* activity and in turn have made an important contribution to this topic [75,76,77,78,79,80,81,82].

**Figure 7:**
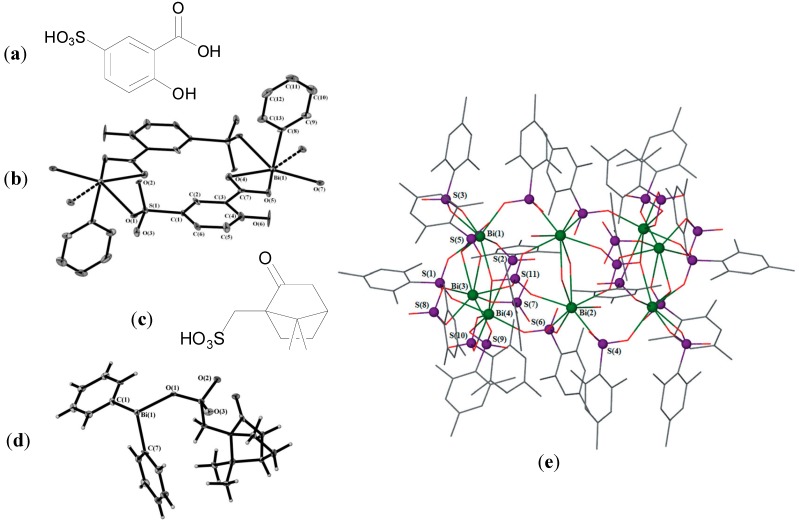
(**a**) Structure of 5-sulfosalicylic acid; (**b**) Structure of [PhBi(HSal)H_2_O]_∞_ showing the basic dimeric unit. Reproduced from [75] with permission from The Royal Society of Chemistry; (**c**) Structure of the *S*-(+)-10-camphoryl sulfonic acid derivative; (**d**) Molecular structure of [Ph_2_Bi(O_3_S-Cam)]_∞_. Reproduced from [76] with permission from The Royal Society of Chemistry; (**e**) Molecular structure of [Bi_8_(O_3_SMes)_20_(SO_4_)_2_(H_2_O)_6_]. Lattice toluene and H atoms omitted for clarity. Reproduced from [78] with permission from The Royal Society of Chemistry.

Andrews initial interest in the synthesis of Bi carboxylates via solvent mediated and solvent free methods lead to the development of polymeric Bi(III) 5-sulfosalicylate complexes. Reaction of 5-sulfosalicylic acid (H_3_Ssal, Figure 7a) with BiPh_3_ gave the heteroleptic carboxylate-sulfonate complexes [PhBi(HSal)H_2_O]_∞_ and [PhBi(HSal)EtOH]_∞_ where 5-sulfosalicylic acid is doubly deprotonated. Reaction of 5-sulfosalicylic acid with Bi(OAc)_3_ gave the homoleptic complex, {[Bi(HSal)(H_2_Sal)(H_2_O)_3_]_2_.2H_2_O}_∞_, where 5-sulfosalicylic acid is found to be both singly and doubly deprotonated [75]. The molecular structure of [PhBi(HSal)H_2_O]_∞_ is shown in Figure 7b. The Bi centre is six-coordinate and the geometry is pentagonal pyramidal. The sterochemically active lone pair is *trans* to the phenyl group found at the apical position. The ethanol complex, [PhBi(HSal)EtOH]_∞_, was determined to be insoluble in aqueous solution whereas the two remaining complexes were remarkably water soluble resulting in solutions of pH 1.5. It is reported that the development of Bi complexes which are not water soluble at low pH is desirable given the links between potentially toxicity, absorption and water solubility. At pH 2 BSS, CBS and RBC are insoluble in aqueous solutions for example. Nonetheless all three complexes had reported MIC values of <6.25 µg/mL against three laboratory strains of *H. pylori* (B128, 251 and 26695). Such activities compare favourably with those of BSS (≤12.5 µg/mL) and 5-sulfosalicylic acid (>25 µg/mL) [75].

Having established that the 5-sulfosalicylic acid complexes had better activity than BSS, Andrews *et al.* were sufficiently encouraged to investigate the structural chemistry and anti-*H. pylori* activity of Bi complexes of mono-functional sulfonic acids [76,78]. Initially three bisphenyl Bi sulfonates were synthesized and characterised, [Ph_2_Bi(O_3_SR)]_∞_ where R is *p*-tolyl, mesityl or *S*-(+)-10-camphoryl (Figure 7c). These complexes were all insoluble in water and aqueous HCl solutions. The molecular structure of [Ph_2_Bi(O_3_S-Cam)]_∞_ is shown in Figure 7d. It consists of polymeric helical chain structure of four coordinate Bi atoms, which bridge two sulfonate O atoms. The geometry is distorted trigonal bipyramidal. These complexes were found to rearrange in solutions of acetone or dmso though to give for example mono-phenyl Bi bis-sulfonates, Bi tris-sulfonates or BiPh_3_. Regardless the presence of the single sulfonato ligands in [Ph_2_Bi(O_3_SR)]_∞_ produced a noteworthy increase in the anti-bacterial activity of the complexes, which had MIC values of ≥6.25 µg/mL as compared to BiPh_3_ (>64 µg/mL) and the inactive sulfonic acids.

Subsequently given the potential toxicity associated with the release of benzene from phenyl bismuth compounds Andrews and co-workers concentrated on the development of Bi tris-sulfonates. Four complexes of general formula, [Bi(O_3_SR)_3_] were synthesized, where R = phenyl, *p*-tolyl, 2,4,6-mesityl or *S*-(+)-10-camphoryl. These complexes exhibited ‘remarkable’ anti-bacterial activity where MIC values as low as 0.049 µg/mL were determined for *H. pylori* strains B128 and 26695. In addition MIC values of 0.781 µg/mL were found for three of the complexes against a clinical *H. pylori* isolate 251, which were significantly lower than the values for BSS (12.5 µg/mL), CBS (12.5 µg/mL), RBC (8 µg/mL) and the corresponding [Ph_2_Bi(O_3_SR)] complexes (6.25 µg/mL), again highlighting the importance of the sulfonate group in relation to the anti-bacterial activity of this class of Bi complexes [78]. Interestingly the structure of [Bi_8_(O_3_SMes)_20_(SO_4_)_2_(H_2_O)_6_].(C_7_H_8_)_7_, which was produced on reaction of MeSO_3_H and BiPh_3_ in toluene was also solved, Figure 7e. This noteworthy structure was described by the authors as a Bi(III)-sulfonate oligomeric cluster, where the eight Bi(III) centres are arranged in a wheel-type structure shielded by the sulfonate ligands [78].

Recently Andrews extended his interest to Bi(III) aminoarenesulfonate complexes [81]. Nine new tris-substituted Bi(III) aminoarenesulfonate complexes of general formula [Bi(O_3_S-R^N^)_3_], where R^N^ = *o*-, *m-* and *p*-aminophenyl, 6-amino-3-methoxyphenyl, 2-pyridyl, *o*-aminonaphthyl, 5-aminonaphthyl, 4-amino-3-hydroxynapthyl and 5-isoquinolinyl. The Bi complexes were found to be insoluble in water and 1 M aqueous HCl solution and also display notable *in vitro* activity against the *H. pylori* strains investigated with MIC values ranging from 0.049 to 12.5 µg/mL [81]. The *o*-, *m-* and *p*-aminophenyl complexes were particularly active against the clinical *H. pylori* isolate 251 with MIC values of 0.049 µg/mL [81], which were lower than the MIC values previously reported for Bi *tris*-sulfonates (0.781 µg/mL) [78]. The identity of the ligand therefore clearly plays a key role in modulating the anti-bacterial effect as further demonstrated by incorporation of the amino functionality onto the sulfonate ligands.

**Figure 8:**
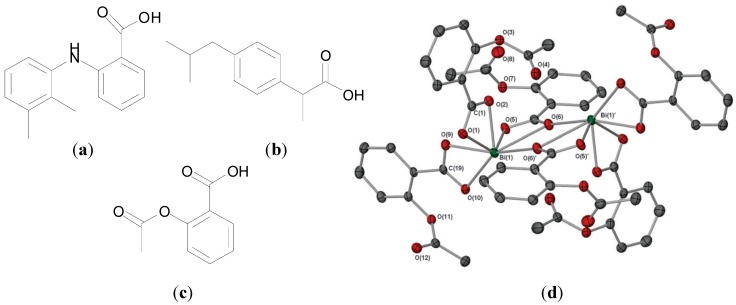
Structures of: (**a**) ibuprofen, (**b**) mefenamic acid and (**c**) acetylsalicylic acid (aspirin); (**d**) Molecular structure of [Bi(asp)_3_]_∞_. Hydrogen atoms have been omitted for clarity. Reproduced from [80] with permission from The Royal Society of Chemistry.

Andrews *et al.* also developed Bi(III) complexes of non-steroidal anti-inflammatories (NSAIDs) [77] and reported the structure and activity of “Bisprin”, a Bi(III) trisacetylsalicylic acid complex, where acetylsalicylic acid is aspirin, Figure 8c. All ten reported NSAID complexes of general formula [BiL_3_]_n_ or [BiL_3_·(H_2_O)]_n_ where L is an NSAID such as ibuprofen or mefenamic acid (Figure 8a,b) were found to exhibit excellent *in vitro* activity against *H. pylori* with MIC values of ≥6.25 µg/mL. NMR, FT-IR and mass spectrometric data indicated that the carboxylate groups chelated Bi(III) in a bidentate fashion [77].

The structure of the tris acetylsalicylate Bi(III) complex, [Bi(O_2_C(C_6_H_4_)OAc)_3_]_∞_ ([Bi(Asp)_3_]), was solved by X-ray crystallography. It adopts a polymeric structure in the solid state. A dimeric section of the polymer is presented in Figure 8d, which shows that the dimer consists of two [Bi(Asp)_3_]. units, where two of the acetylsalicylate moieties per Bi(III) centre are bidentate chelating, and the units are joined by O atoms from two unsymmetrical bridging carboxylates. The coordination number is 7 in the dimer, but 8 once polymerization is considered [80]. The complex exhibited similar *in vitro* activity as the previously reported Bi NSAID complexes against *H. pylori* strains investigated (B128, 251 and 26695) with MIC values of 6.25 µg/mL [80].

**Figure 9:**
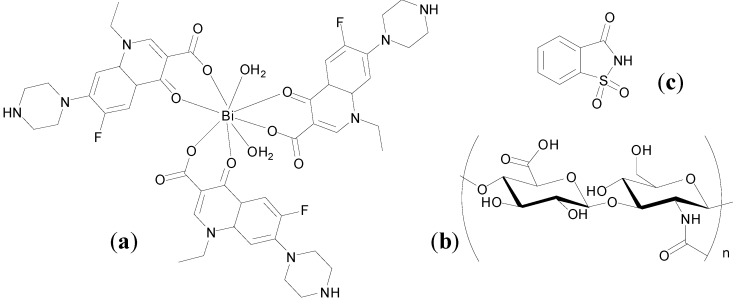
(**a**) Proposed structure of Bi(III) norfloxacin complex; (**b**) Structure of hyaluronic acid; (**c**) Structure of saccharin.

Novel Bi(III) complexes of fluoroquinolones, a family of broad spectrum antibiotics, which also possess good anti-*H. pylori* activity, were developed by Shaikh and co-workers [83]. The complexes were characterised by molar absorptivity determination, differential scanning calorimetry, thermogravimetric analysis, Karl-Fischer aquametry, elemental analysis and FT-IR spectroscopy. The proposed structure of the Bi(III) norfloxacin complex is shown in Figure 9a as a representative example. All of the complexes developed exhibited better activity than their respective fluoroquinolone ligands against 16 clinically isolated strains of *H. pylori*. Significantly the complexes were also found to be active against some of the fluoroquinolone resistant strains investigated with MIC values of 1–4 mg/L (1–4 µg/mL).

Two publications from China report on Bi(III) complexes of a glycosaminoglycan [84] and polysaccharides [85]. Hyaluronic acid (HA, Figure 9b), is a naturally occurring glycosaminoglycan, which consists of repeating disaccharide units of *N*-glucosamine and D-glucoronic acid. It is highly negatively charged under physiological conditions and interacts readily with cations and has been demonstrated to bind to Cu(II), Ag(I), Au(III) and Fe(III) for example. Bi(III) complexes of high (1240 kDa), medium (675 kDa) and low molecular weight HA (240 kDa) were synthesized and characterised by elemental analysis, FT-IR, NMR and CD spectroscopy, X-ray diffractometry, X-ray photoelectron spectroscopy and thermogravimetric analysis. It was proposed that Bi(III) binds to the hydroxyl, carboxylate and amino groups of HA. All Bi(III) HA complexes regardless of molecular weight exhibited similar activity as CBS (MIC = 5 µg/mL) against three strains of *H. pylori* (NCTC11637, 26695 and J99).

Two novel Bi(III) *H. erinaceus* polysaccharide (HEP) complexes were reported recently. *H. erinaceus* is an edible mushroom with well-known medicinal properties [85]. The bodies of *H. erinaceus* are used as home remedies to treat gastric and duodenal ulcers amongst other diseases. Two polysaccharides of 197 and 20 kDa were isolated from *H. erinaceus* and reacted with Bi(NO_3_)_3_ to give HEP complexes, which were characterised by elemental analysis, FT-IR and CD spectroscopy, SEM, AFM, X-ray diffractometry and thermogravimetric analysis [85]. The two HEP were shown to exhibit modest activity against the *H. pylori* strain investigated (NTCC11637) with MIC values of ≥160 µg/mL. The Bi HEP complexes exhibited good activity though with MIC values of 20 µg/mL, which are comparable with the activity of CBS in the same study. In addition the authors highlighted that the Bi HEP complexes developed have a lower Bi content than CBS [85].

The activities of the Bi(III) HA and HEP complexes both exhibited similar activity as CBS, the standard used in their studies. The use of high molecular weight glycosaminoglycans and polysaccharides is an interesting approach though neither authors speculate on the potential physiological impact of using such high molecular weight molecules on absorption for example. In addition characterisation of Bi(III) complexes of mono- or disaccaharides or derivatives of both would surely provide an insight into the exact nature of the interactions of Bi(III) and carbohydrates. Andrews has published reports on Bi(III) complexes of the artificial sweetener saccharin (Figure 9c) [86], and acetosulfame and cyclamic acid [82].

There are an increasing number of excellent publications on novel Bi(III) complexes as potential agents for the treatment of *H. pylori*. These publications feature complexes with well-defined, fascinating and varied structures and excellent anti-*H. pylori* activity. Nonetheless in depth mechanistic studies and in turn *in vivo* studies in relation to some of the more promising compounds are highly anticipated. Perhaps we should encourage/ challenge our colleagues in micro/molecular biology to take a more active interest in elucidating the mechanisms of action of Bi-based *H. pylori* drugs. In summary, eradication of *H. pylori* is an important goal for the global scientific community given its association with dyspepsia, peptic ulcer disease and gastric cancer. Further insight into the mechanism of action of Bi(III) anti-bacterial effects against *H. pylori* and ability to heal ulcers will certainly better inform the rational development of novel, effective and well-defined Bi-based drugs for the treatment of gastrointestinal disorders.

## 3. Anti-Microbial

In addition to *H. pylori*, Bi compounds have been effectively used to treat a host of bacterial associated infections such as syphilis (e.g., potassium bismuth tartrate, bismuth quinine iodide and iodobismitol), colitis (bismuth subnitrate, bismuth citrate), diarrhea (BSS and bismuth nitrate) and wound infections (bismuth oxide) [14].

BSS for example is well-known for its preventative and therapeutic properties in relation to diarrheal disorders. It has been demonstrated to exhibit *in vitro* anti-bacterial activity against enterotoxigenic *E*. *coli*, which is the principal bacterial cause of diarrhea in the developing world and so called “travelers’ diarrhea”. It has been proposed that BSS has the capacity to significantly reduce the toxin secretory activity of *E. coli*. In addition CBS and BSS have activity against another enteropathogen, C. *difficile*; CBS has an *in vitro* minimum inhibitory concentration of 90% of growth (MIC_90_) of 128 µg/L [87], while BSS exhibited noteworthy activity in an *in vivo* hamster model of *C.* *difficile* colitis [88].

Interestingly, Sox and Olson reported that BSS could bind to *E. coli (ATCC 10536)*, *S. typhimurium* and *S. aureus*. In turn binding was linked with bactericidal activity against *E. coli* and *S. typhimurium* though not against *S. aureus* [89]. Interestingly intracellular ATP levels in the *E. coli* strain were found to drop to 10% of initial levels, 30 min post exposure and extracellular ATP levels increased rapidly. It is proposed that exposure to BSS could affect membrane integrity and/or intracellular ATP synthesis. It is also noteworthy that: (i) pretreatment of BSS with albumin decreased the rate of binding and killing of *E. coli*; (ii) the activity of BSS against *E. coli* was much more rapid at pH 3 as opposed to pH 7; (iii) salicylates also possess anti-bacterial activity and therefore may also contribute to the activity of BSS [14,89].

The biochemical targets of Bi-based compounds have been studied much more extensively for *H. pylori* than other bacteria. Nonetheless it is reasonable to assume that bacteria in general share some key biological targets of Bi-based compounds.

Given the well-established anti-bacterial activity of Bi-compounds, there has been somewhat of a resurgence of interest in the development of novel Bi-based compounds as anti-bacterial agents. In contrast there are few if any historical reports in relation to the anti-fungal activity of Bi-based compounds and in turn limited evidence in relation to potential mechanisms of action. Nonetheless there have been some recent reports investigating the anti-fungal activity of Bi-based compounds though typically accompanying anti-bacterial data.

**Figure 10:**
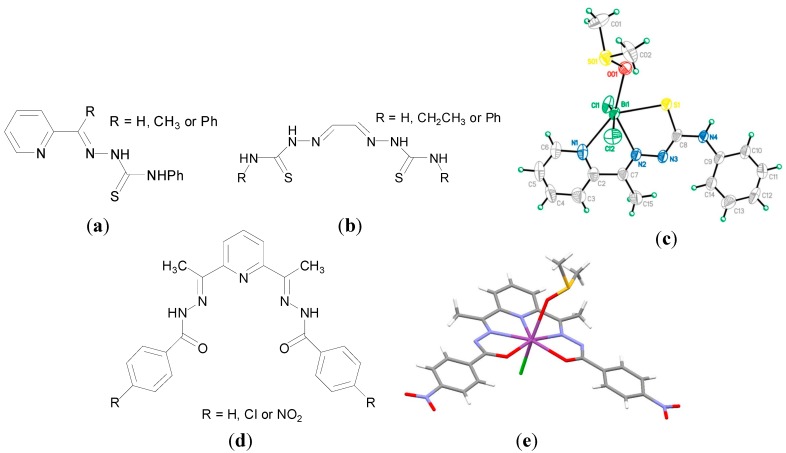
(**a**) General structure for pyridin-2-yl-derived 4-phenylthiosemicarbazones; (**b**) General structure for glyoxaldehyde bis(thiosemicarbazones); (**c**) Structure of [Bi(_2_Ac_4_Ph)(dmso)Cl_2_]. Reprinted with permission from [90]. Copyright (2012) John Wiley and Sons; (**d**) general structure for 2,6-diacetylpyridine bis(benzoylhydrazones) and (**e**) Molecular structure of [Bi(AcpNO_2_Ph)(dmso)Cl] (CCDC no.859622); Colour code:  C, grey; H, white; O, red; N, blue; S, yellow; Cl, green; Bi, purple [95].

There has been considerable interest in the potential biological activity of Bi(III) thiosemicarbazone complexes. Thiosemicarbazones, derived from condensation reactions between thiosemicarbazides and aldehydes or ketones, have rich coordination chemistry and are reported to have anti-parasitic, anti-bacterial and anti-cancer properties [90]. In addition it has been previously demonstrated that the anti-microbial activity of thiosemicarbazones can be enhanced on coordination to Sn, Cu and Ga [90]. Lessa *et al.* reported a family of Bi(III) thiosemicarbazones and bis(thiosemicarbazone) complexes as potential anti-bacterial agents. The complexes developed exhibited good anti-bacterial activity, where both the mono- and bis(thiosemicarbazone) Bi(III) complexes were better anti-bacterial agents than their corresponding free ligands (Figure 10a,b) against the *Gram*-positive bacteria; *S. aureus*, *S. epidermidis* and *E. faecalis.* The crystal structure of [Bi(2Ac4Ph)(dmso)Cl_2_]. (H2Ac4Ph = 1-(pyridin-2-yl)ethanone 4-phenylthiosemicarbazone, Figure 10c, R = CH_3_) was solved, exhibiting the monoanionic coordination mode of the thiosemicarbazone via the N_py_, N_imine_ and S. The increase in activity on complexation was particular evident against *S. aureus*, where for example the MIC for H2Fo4Ph (pyridine-2-carbaldehyde 4-phenylthiosemicarbazone, Figure 10a, R = H) of 190 µM decreased to 6.1 µM for [Bi(2Fo4Ph)Cl_2_]. The MIC for tetracycline hydrochloride against *S. aureus* was found to be 0.3 µM though. Only one of the complexes tested, [Bi(2Fo4Ph)Cl_2_], exhibited enhanced activity as compared to its free ligand against the *Gram*-negative bacteria, *P. aeruginosa*.

Lessa *et al.* proposed that an increase in solubility and bioavailability of Bi on coordination was key to the observed activity [90]. There are a number of additional reports in relation to novel bismuth thisosemicarbazone complexes, which were investigated not only as anti-bacterial agents but also anti-cancer agents [19,91,92,93,94]. These complexes feature in Section 5, where only their anti-cancer activity is discussed.

A report also from the same group in Belo Horizonte, Brazil discussed the development of a series of Bi(III) complexes of 2,6-diacetylpyridine bis(benzoylhydrazone) derivatives (Figure 10d) and their anti-microbial activity against *S. aureus*, *E. facecalis*, *S. epidermidis*, *P. aeruginosa and C. albicans*. Hydrazone derivatives are known to possess anti-microbial and anti-tubercular activities [95], though in this study the ligands investigated were found to be inactive against the panel of microorganisms investigated. In contrast and in general upon coordination to Bi(III) the anti-microbial activity of the hydrazone derivatives was enhanced against the *Gram*-positive bacteria though less notably against the *Gram*-negative bacteria (*P. aeruginosa*). Significantly the Bi(III) complexes were more active than their Sb(III) analogues and the Bi(III) hydrazone complex, [Bi(HAcpNO_2_Ph)Cl_2_], where H_2_AcpNO_2_Ph is 2,6-diacetylpyridine bis(para-nitrobenzoylhydrazone), was found to be more active than tetracycline against *S. aureus* [95]. The X-ray crystal structure of [Bi(HAcpNO_2_Ph)(dmso)Cl] (Figure 10e), was solved on recrystallization of [Bi(HAcpNO_2_Ph)Cl_2_]. from acetone/dmso, again highlighting Bi(III) affinity for dmso. The anti-fungal activity of the Bi(III) complexes against *C. albicans* were also better than their Sb(III) analogues. The Bi(III) hydrazone complex, [Bi(HAcPh)Cl_2_], where H_2_AcPh is 2,6-diacetylpyridine bis(benzoylhydrazone), (MIC: 44 µM) exhibited better activity than fluconazole (MIC: 59 µM), a well-known anti-fungal agent [95].

Bi-based ciprofloxacin compounds were developed by Turel and co-workers [96,97]. Ciprofloxacin is a second generation fluoroquinolone antibiotic and development of Bi-based ciprofloxacin compounds was therefore an interesting strategy for the targeting of anti-microbial resisitance [96,97]. Two Bi(III) ciprofloxacin (cf) compounds were reported; (cfH_2_)(cfH)[BiCl_6_]·2H_2_O and (cfH_2_)_2_[Bi_2_Cl_10_]·2H_2_O (cf = 1-cyclopropyl-6-fluoro-4-oxo-7-(piperazin-1-yl)-quinoline-3-carboxylic acid) [96,97]. No bonding between the quinolone and Bi(III) was observed though as the quinolones are either singly protonated (cfH) at piperazine N or doubly protonated (cfH_2_) at the carbonyl O and piperazine N. In (cfH_2_)(cfH)[BiCl_6_]·2H_2_O the six chloride ions coordinate Bi(III) with octahedral geometry to give [BiCl_6_]^3−^ anions, whereas in (cfH_2_)_2_[Bi_2_Cl_10_]·2H_2_O the Bi(III) is coordinated by chloride ions to give a centrosymmetric [Bi_2_Cl_10_]^4−^ anion. Antimicrobial testing of (cfH_2_)(cfH)[BiCl_6_] against a panel of *Gram*-negative and *Gram*-positive bacteria and fungi was undertaken. In summary the compound exhibited similar activity as cf·HCl against the *Gram*-negative (*E. coli*, *P. aeruginosa*) and *Gram*-positive bacteria (*S. aureus*, *E. facecalis*, *B. Subtilis*, *B. cereus*) and no activity against the fungi (*C. albicans*, *T. mentagrophytes* and *A. niger*) investigated [96,97]. Both (cfH_2_)(cfH)[BiCl_6_] and (cfH_2_)_2_[Bi_2_Cl_10_] were also demonstrated to have similar activity as cf.HCl against *S. viridians*, *Enterococcus* sp. and *S. haemolyticus G*, which are resistant to ciprofloxacin*.* The authors appropriately discuss the potential impact on speciation of dissolving their compounds in DMSO and increasing the pH on diluting the compound with media and serum for example. BiCl_3_ for example is known to form BiOCl readily in aqueous solutions and Bi(III) dimethyl sulfoxide complexes will also potentially form when Bi(III) complexes are dissolved in dmso as evidenced by the structure in Figure 10c,e [95].

**Figure 11:**
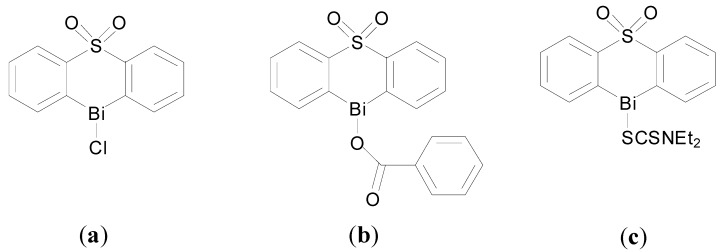
Structures of heterocyclic organobismuth(III) compounds derived from diphenyl sulfone. (**a**) Chlorobismuthane; (**b**) A carboxylate bismuthane; (**c**) A dithiocarbamate bismuthane.

Murafuji and co-workers have developed heterocyclic organobismuth compounds as anti-fungal agents [98,99,100]. They initially identified a family of halobismuthanes derived from diphenyl sulfone as exhibiting promising anti-fungal activity against *S. cerevisiae*. Interestingly the X-ray crystal structure of the chlorobismuthane (Figure 11a), reveals the asymmetric unit contains two independent molecules with different coordination geometries at the Bi centre: (i) five coordinate Bi centre geometry through intramolecular Bi-O and intermolecular Bi-Cl interactions and (ii) seven coordinate Bi centre through intramolecular and intermolecular coordinated Bi-O bonds, which is an unusual geometry for organobismuth(III) compounds. Inhibitory effects were linked with the Lewis acidity of the Bi(III) centre which was proposed to be the active site [100].

Subsequent investigations of the effect of introducing substitutents onto the diphenyl sulfone scaffold and replacement of the chloro group attached to the Bi centre, were undertaken [98,99]. The findings suggest that in general there is an inverse relationship between lipophilicity of this family of compounds and anti-fungal activity; the higher the lipophilicity (ClogP values) the lower the activity of the halobismuthanes. Nonetheless a series of organobismuth(III) carboxylates, e.g., Figure 11b, display similar activity to chlorobismuthane (Figure 11a) even though they have higher ClogP values. Organobismuth(III) dithiocarbamate analogues, Figure 11c, were inactive, which was attributed to the lowered Lewis acidity due to the lower electronegativity of the sulphur atom bound to bismuth and also the high ClogP values associated with these compounds [98].

A subsequent study of the influence of varying the structure of the carboxylate ligand on lipophilicity and anti-fungal activity suggested that the lipophilicity of the carboxylate compounds had no bearing on anti-fungal activity. Therefore the authors propose that the organobismuth(III) carboxylates separate inside yeast cells into a cationic heterocyclic bismuth scaffold and ionic carboxylate moiety with the bismuth scaffold playing the key role in anti-fungal activity [99].

Bi-based compounds are and have been successfully used to treat bacterial infections [101]. There has been recent interest in this area and encouraging results. Surprisingly though, there are fewer reports of novel Bi-based compounds as potential anti-bacterial agents in the literature relative to other metal-based compounds of gold and silver for example. Perhaps the anti-bacterial properties associated with Bi have been somewhat “forgotten” or the lack of solid evidence in relation to mechanism of action and the identification of concrete biomolecular targets is slowing progress in this area. There is a definite lack of reports in relation to Bi-based drugs as anti-fungal agents with little or no publications investigating possible anti-fungal mechanisms of action of Bi compounds. Accordingly exciting opportunities and challenges exist in this area.

## 4. Anti-Leishmaniasis

Leishmaniasis is a group of diseases caused by protozoan parasites of the *Trypanosomatidae* family and typically contracted by the bite of an infected female sand fly. In Asia, Africa and Southern Europe it is transmitted by the genus *Leishmania* whereas in North and South America it is transmitted by the genus *Phlebotomus* [102,103]. The epidemiology of the contracted disease depends on the strain and characteristic of the parasite species as well as other contributing factors. It is reported to be prevalent in 98 countries worldwide, where over 350 million people are at risk of contracting the disease. 12 million are reported to have leishmaniasis with a further estimated two million new cases occurring every year [104].

There are three main forms of the disease; *visceral*, *cutaneous* and *mucocutaneous*. *Visceral*, caused by *L. donovani* and *L. infantumis* is the most dangerous form of the disease and if not treated promptly and completely can result in death very quickly [105]. It is reported that 300,000 cases result in about 20,000 deaths every year for example. The *cutaneous* and *mucocutaneous* forms of the disease can be devastating; in the cutaneous form ulcers appear at the site of the bite, often resulting in scarring [106], whereas in the *mucocutaneous* form partial or complete destruction or depletion of the cutaneous and subcutaneous tissue occurs.

**Figure 12:**
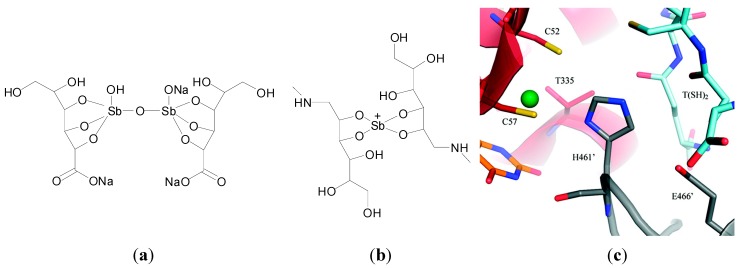
Structures of (**a**) sodium stibogluconate and (**b**) meglumine antimoniate; (**c**) Overall view of the catalytic cleft of trypanothione reductase (TR) with Sb(III) from T. Infantum (PDB code 2W0H). The residues involved in trypanothione reduction are indicated as sticks [109]. The trypanothione substrate, modelled on the basis of the *T. cruzi* TR structure (PDB code 1BZL), is also indicated as sticks and coloured cyan. Reprinted with permission from [109]. Copyright 2012 American Chemical Society.

Antimony (Sb)-based drugs have been used for the treatment of leishmaniasis. Tartar emetic (potassium antimony tartrate), a Sb(III) compound for example, developed at the start of the 20th century, was initially used and increased survival rates but was highly toxic to the patient [107]. Today however pentavalent substitutes such as pentosam (sodium stibogluconate) and glucantime (meglumine antimoniate) [108] (Figure 12a,b) are used as first-choice anti-leishmanial drugs in many countries due to their lower toxicity and more efficient therapeutic indices [14].

The exact mode of action and site of action of the Sb-based drugs is for the most part unknown. It is widely accepted that the pentavalent Sb(V) is reduced to Sb(III) in the macrophages and parasitic cells by different thiols including cysteine, cysteine glycine and trypanothione [63,110]. Proposed biomolecular targets include the trypanothione synthase and reductase system. Trypanothione is a form of gluthatione, specific to the parasite in question. Trypanothione synthase, (TS), conjugates glutathione and spermidine to form trypanothione (N1-N8-bis(glutathionyl)spermidine), while trypanothione reductase (TR), keeps the molecule in its reduced form. The TS and TR systems are essential for survival of the parasites and the *Typanosomatidae* family as they rely on it to replace the redox functions of systems such as the glutathione reductase system present in other organisms. Crystal structures revealed the Sb binding site in TR and thus provided the molecular basis for antimonial inhibition of TR. Upon reduction of TR by NADPH, a disulphide bridge associated with two cysteine residues (Cys52 and Cys57) separates to form two thiolate groups, just 4.4 Å apart. The Sb(III) ion is coordinated in a distorted tetrahedral geometry by the two aforementioned cysteinate sulphurs, one threonine (Thr335) and one histidine (His461) from a second TR that forms a dimer complex. Comparison of the reduced TR Sb(III) NADPH complex structure with trypanothione bound TR from *L. cruzi* shows that Sb(III) is binding at the catalytic site of the enzyme (Figure 12c). Sb(III) therefore works by preventing interaction between the two sulphur atoms of the cysteine residues with His461, which acts as a proton exchanger in the reduction mechanism associated with glutathione reductase [111]. Ultimately the parasite’s ability to buffer oxidative stress is thought to be decreased [112].

An alternative proposal to explain the anti-leishmanial activity of Sb(III) has been published by Frezard *et al.* suggesting that Sb(III) targets important zinc finger binding domains responsible for many regulatory functions, e.g., DNA recognition, RNA packaging, protein folding and assembly, transcriptional activation, cell differentiation, growth and apoptosis. They showed that Sb(III) can compete with zinc(II) for binding motifs found in Leishmanial parasites and proposed that this competitive binding may be responsible for the pharmacological activity of Sb drugs [113].

Resistance to Sb(V) first line drugs, as well as other contributing factors such as harsh dosage regimens and cardio- and hepato-toxicity have driven research to find novel drug compounds which could be used to treat leishmaniasis [82]. Given Bi close proximity in the periodic table and similar biological chemistry to Sb there is potential for Bi drugs to have similar biomolecular targets. Therefore Bi could be an ideal candidate for the development of novel metal-based drugs in this field. There are only a few, nevertheless important, examples of Bi-based compounds, which have been developed and evaluated as potential anti-leishmanial drugs [82,110,114,115].

**Figure 13:**
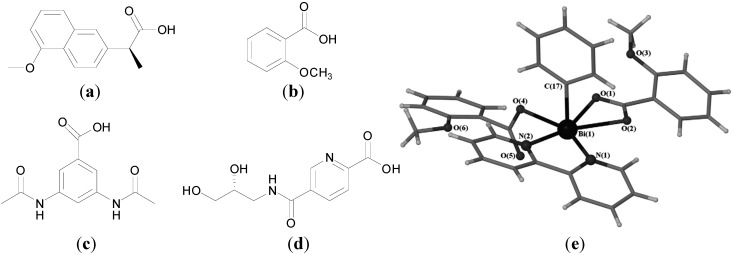
Structures of (**a**) naproxen, (**b**) *o*-methoxybenzoic acid, (**c**) 3,5-diacetamido-benzoic acid and (**d**) 5-[(*R*/*S*)-2,3-dihydroxypropylcarbamoyl]-2-pyridinecarboxylic acid; (**e**) Molecular structure of [PhBi(o-MeOC_6_H_4_CO_2_)_2_(bipy)]. Reprinted with permission from [110]. Copyright (2011) Elsevier.

Andrews, who has published extensively in relation to Bi compounds with activity against *H. pylori*, is also interested in developing Bi compounds with anti-leishmanial activity [82,110]. The activity of four NSAIDs, naproxen (Figure 13a) mefenamic acid, ketoprofen and diflunisal and their corresponding homoleptic tris-carboxylato Bi(III) complexes, of general formula [BiL_3_]_n_ and which were previously investigated for anti-*H. pylori* activity [77], were investigated against *Leishmaniasis major* promastigotes and human primary fibroblast cells for 48 h. Both groups showed activity only at the highest concentration tested (500 µg/mL) against *L. major* parasites, which is considered too high to be of practical use. A significant difference in the activity of naproxen and its tris-carboxylato Bi(III) complex was observed though, supporting a potential anti-leishmanial role for Bi. The Bi complexes were also more toxic towards human fibroblasts than the free acid also indicating that bismuth has a role in human cell toxicity [110].

In addition Bi(III) complexes of substituted benzoic acids; *o-* and *m*-methoxybenzoic acid (Figure 13b) *o*-nitrobenzoic acid, 3,5-diacetamidobenzoic acid (Figure 13c), and 5-[(*R/S*)-2,3-dihydroxypropyl carbamoyl]-2-pyridinecarboxylic acid (Figure 13d), were reported. Choice of ligand and reaction conditions dictated complex type produced; [BiL_3_(H_2_O)_3_], [PhBiL_2_], [PhBiL_2_(bipy)] (bipy-2,2-bipyridine), [PhBiL_2_(H_2_O)_2_] and [PhBiL_2_(H_2_O)]. Interestingly the free substituted carboxylic acids (examples **5**–**9**) displayed no activity against *L. major* parasites but their corresponding Bi derivatives were quantitatively toxic against the parasite even at low concentrations (1.95–200 µg/mL). The *o*-methoxybenzoic acid complex, [PhBi(o-MeOC_6_H_4_CO_2_)_2_(bipy)], possessed the lowest IC_50_ of 1.9 µg/mL. Its structure was solved by X-ray crystallography (Figure 13e). It is six-coordinate with distorted pentagonal pyramidal geometry. The stereochemically active lone pair is found *trans* to the phenyl group found at the apical position. When tested for potential human toxicity, against the fibroblast cell line, the Bi complexes in general were significantly more toxic than both the free substituted benzoic acids and the previously discussed Bi NSAID complexes.

Andrews and co-workers recently reported a series of Bi(III) β-thioxoketonate complexes as anti-leishmanial agents. They hypothesised that Bi complexes with a more thermodynamically stable Bi-S bond would be less labile than for example a carboxylate analogue and in turn possess improved hydrolytic stability which should positively influence purity, reproducibility and activity in biological systems [82]. Though transition metal and p-block metal, including Bi, β-thioxoketonate complexes have been reported, the biological chemistry of the ligands or complexes were not explored [82].

**Figure 14:**
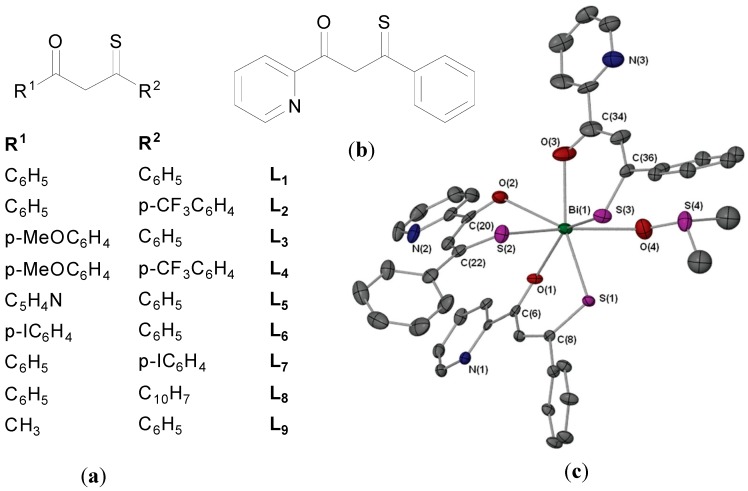
(**a**) Structures of β-thioxoketones; (**b**) Structure of L_5_; (**c**) Molecular structure of [Bi{C_5_H_4_NC(=O)CHC(=S)C_6_H_5_}_3._dmso]. Reproduced from [82] with permission from The Royal Society of Chemistry.

Nine different β-thioxoketones complexes of formula R^1^C-(=O)CH_2_(=S)R^2^ (Figure 14a), and their Bi complexes of formula [Bi{R^1^C(=O)CHC(=S)R^2^}_3_] were synthesized. The structure of [Bi{C_5_H_4_NC(=O)CHC(=S)C_6_H_5_}_3_.dmso] was solved on attempted recrystallization of [Bi{C_5_H_4_NC(=O)CHC(=S)C_6_H_5_}_3_] in DMSO (Figure 14c). The complex is 7-coordinate and has disordered pentagonal bipyramidal geometry. As expected the β-thioxoketonate ligand chelates the Bi(III) centre in a bidentate fashion via the O and S atoms.

The bismuth derivatives, free acid and BiPh_3_ were tested for their *in vitro* toxicity activity against *L. major* promastigotes and primary human fibroblasts. The β-thioxoketones were selectively toxic to *L. major* promastigotes as opposed to the primary human fibroblasts. L_1_ (Figure 13a), for example had similar activity as the antibiotic, Amphotericin B, with *ca*. 80% parasite kill at 25 µM (6 µg/mL). In contrast and surprisingly the Bi(III) thioxoketonates were in general less toxic than their free ligands. The L_5_ Bi complex, [Bi{C_5_H_4_NC(=O)CHC(=S)C_6_H_5_}_3_]. (B5) was the most active of the series with *ca*. 80% parasite kill at 50 µM (46 µg/mL). Though B5 was less toxic to the fibroblasts compared against the *L. major* promastigotes, in general the Bi complexes displayed little or no selectivity, which would suggest that they are not suitable candidates for the treatment of Leishmaniasis. Lipophilicity, stability and low lability were highlighted as factors that may be important in determining cellular uptake and interactions with biomolecular targets.

These two contrasting studies highlight that Bi does have anti-leishmanial properties though such properties are modulated by ligand choice as well as factors such as lipophilicity and stability. The development of compounds with selective anti-leishmanial activity is the challenge.

**Figure 15:**
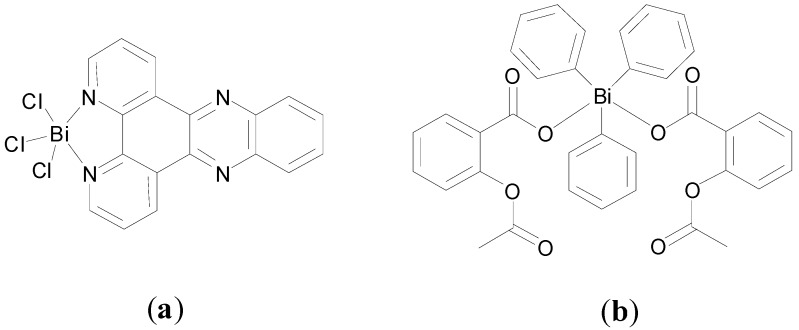
Proposed structures of (**a**) [Bi(dppz)Cl_3_] and (**b**) [Ph_3_Bi(AcSA)_2_].

Demicheli and co-workers are also interested in the development of Bi-based compounds as anti-leishmanial agents [114,115]. In 2012 they reported Sb(III) and Bi(III) complexes of dipyrido[3,2-a:2',3'-c]phenazine (dppz), [Sb(dppz)Cl_3_] and [Bi(dppz)Cl_3_] (Figure 15a) which were characterized by FT-IR and NMR spectroscopy, elemental analysis and X-ray crystallography (Sb complex only). These complexes, along with the free ligand dppz were tested against wild type (WT) and Sb resistant (SbR) strains of *Leishmania infantum chagasi* and *Leishmania amazonensis,* which are associated with visceral and cutaneous Leishmaniasis respectively.

Dppz showed intrinsic activity against both the WT and SbR strains investigated with IC_50_ varying from *ca*. 0.8 to 2 μM. As expected the Sb(III) and Bi(III) complexes showed a higher activity against the same strains (*ca*. 0.6–1 μM), indicating a role for the Sb(III) and Bi(III) in the activity of the complexes. Interestingly the Sb(III) and Bi(III) compounds, which had very similar activity, were at least 77 and 2400 times more active than tartar emetic against the WT and SbR strains respectively. Both complexes are 80 times more active than the trivalent BiCl_3_ and SbCl_3_ compounds than the WT strains also. This was interesting to note as this suggests that the metal alone is not sufficient to have anti leishmaniasis activity but perhaps as proposed improves the activity of dppz through complexation [115].

It was hypothesised that the leishmanicidal activity of the dppz complexes may be via dppz intercalation at the parasites DNA and/or positive modulation of the lipohilicity/hydrophilicity of dppz on complexation. In this study the less lipophilic (more hydrophilic) the compound the better activity against leishmaniasis for example [115]. A similar observation in relation to lipophilicity was observed in Andrews recent study in relation to the activity of the β-thioxoketones, which were generally more active but less lipophilic than their Bi(III) complexes [82].

Significantly the cytotoxicity studies of the Bi(III) and Sb(III) complexes against mouse peritoneal macrophages (MPM) showed toxicity occurs at a 6-fold higher level as compared to the active concentration against leishmaniasis promastigotes, indicative of the potential selectivity of these compounds.

In a very recent study, two novel triphenyl Sb(V) and two triphenyl Bi(V) complexes of benzoic acid derivatives and their respective free ligands were screened for activity against *L.* *amazonensis* and *L.* *infantum* promastigotes. Complexes of the type [Ph_3_ML_2_] were developed where HL is acetylsalicylic acid (aspirin, AcSAH) or 3-acetoxybenzoic acid (3-AcBAH) and characterised by FT-IR and NMR spectroscopy, elemental analysis and X-ray crystallography (Sb complex only) [114]. The proposed structure for [Ph_3_Bi(AcSa)_2_] is shown in Figure 15b.

As per Table 1 the benzoic acid derivatives show no activity against the leishmaniasis strains at 48 h treatment. The novel organo Bi(V) benzoic acid derivative complexes (2.9–8.6 µM IC_50_ range) were more active than the corresponding Sb(V) complexes (8.9–30.7 µM IC_50_ range) thought both were notably more active than tartar emetic (83–100 µM IC_50_ range). Significantly the Sb and Bi salts, Ph_3_SbCl_2_ and Ph_3_BiCO_3_ were at least as active as the complexes and in fact Ph_3_BiCO_3_ was the most active compound tested, suggesting that the ligands probably dissociate from the complexes prior to them exerting their activity. 

**Table 1:** Inhibitory concentrations of triphenyl Sb(V) and triphenyl Bi(V) complexes of acetylsalicylic acid and 3-acetoxybenzoic acid, acetylsalicylic acid, 3-acetoxybenzoic acid and metal salts against *L. amazonensis* and *L. infantum* species promastigotes at 48 h [114].

Compounds	*L. infantum* Strain	*L. amazonensis* Strain
	IC_50_ (μM) ± SEM	
Acetylsalicylic acid (AcSAH)	8.99 × 10^3^ ± 1.89 × 10^3^	2.18 × 10^6^ ± 0.12 × 10^6^
Ph_3_Sb(AcSA)_2_	13.3 ± 0.74	30.7 ± 3.43
Ph_3_Sb(3-AcBA)_2_·CHCl_3_	12.2 ± 0.91	8.9 ± 0.36
Ph_3_SbCl_2_	13.2 ± 2.03	9.3 ± 0.26
3-Acetoxybenzoic acid (3-AcBAH)	3.19 × 10^7^ ± 0.55 × 10^7^	1.40 × 10^7^ ± 0.12 × 10^6^
Ph_3_Bi(AcSA)_2_	8.6 ± 1.36	8.5 ± 0.56
Ph_3_Bi(3-AcBA)_2_	4.0 ± 0.39	2.9 ± 0.17
Ph_3_BiCO_3_	1.1 ± 0.37	2.7 ± 0.34
Tartar emetic	100 ± 3	83 ± 1

Cytotoxicity against mammalian cells was investigated by means of MTT assay on mouse peritoneal macrophages (MPM). The organo Sb(V) complexes were found to be approximately 10 fold less toxic than organo Bi(V) complexes, and are hence more selective to the leishmanial strains in this study.

Leishmaniasis is prevalent in many countries worldwide. It is a devastating disease but one which is curable once treated correctly and rapidly. Nonetheless resistance, compliance and toxicity are factors associated with current treatment options. Therefore there has been recent interest in developing new Bi-based drugs which can successfully treat leishmaniasis and Bi compounds with promising activity have been reported. The continued development and evaluation of novel Bi compounds and determination of Bi effect on the TS and TR systems and other potential leishmanial biomolecular targets, will certainly further the cause of Bi- compounds as potential anti-leishmanial agents.

## 5. Anti-Cancer

Cancer is a major cause of death and disease worldwide and according to WHO cancer related deaths are predicted to rise to over 21 million by 2030 [116]. Over the past 30 years platinum compounds have played a very important and well documented role in treating cancer. In spite of the clinical and commercial success of platinum compounds there are well-known drawbacks associated with their use such as toxicity, limited activity and resistance [5]. Nonetheless a vast amount of research has been undertaken to date in relation to non-platinum-based compounds as anti-cancer agents. Radiometals aside, it may therefore be surprising to note that arsenic is the only other metal whose compounds are approved for the direct treatment of cancer, (acute promyelocytic leukaemia, APL) [3]. It may also be surprising given the success of As_2_O_3_ in the treatment of APL, that there is a lack of reports in relation to the development of novel Bi-based anti-cancer chemotherapeutics. A selection of contributions to this area is outlined below.

**Figure 16:**
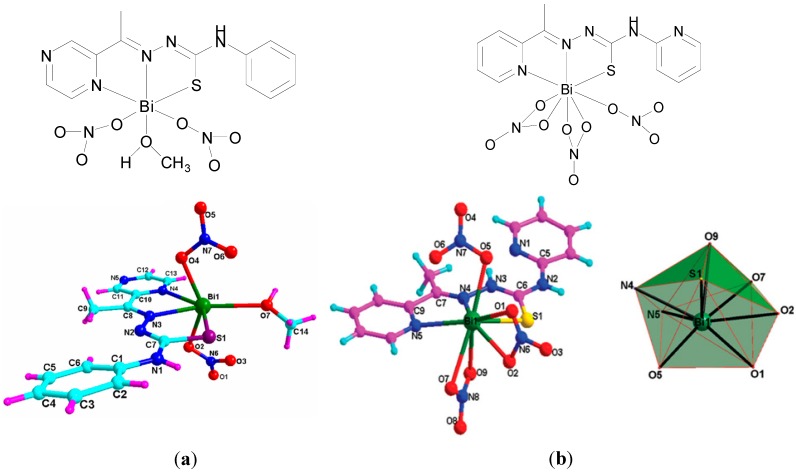
Molecular structures of (**a**) a Bi(III) complex of 2-acetylpyrazine *N*(4)-phenylthiosemicarbazone (HL), [Bi(L)(NO_3_)_2_(CH_3_OH)]. Reprinted with permission from [92]. Copyright (2012) Elsevier and (**b**) a Bi(III) complex of 2-acetylpyridine, *N*(4)-pyridyl thiosemicarbazone (HL), [Bi(HL)(NO_3_)_3_] and polyhedron showing dodecahedral geometry around the bismuth atom of the asymmetric unit. Reproduced from [91] with permission from The Royal Society of Chemistry.

A number of recent publications from Henan University, Kaifeng, China contribute to the story in relation to bismuth thisosemicarbazone or thiocarbonohydrazone complexes as anti-cancer agents [19,91,92,93,94]. As previously mentioned, in relation to the development of Bi-based anti-bacterial agents, thiosemicarbazones, have rich coordination chemistry and are reported to have anti-parasital, anti-bacterial and anti-cancer properties. Ming-Xue Li *et al.* developed a Bi(III) complex of 2-acetylpyrazine *N*(4)-phenylthiosemicarbazone (HL), [Bi(L)(NO_3_)_2_(CH_3_OH)]. The structure of this complex was solved by X-ray crystallography (Figure 16a) [92]. This complex is 7-coordinate, accounting for the inert electron pair, and possesses pentagonal bipyramidal geometry. The tridentate monodeprotonated thiosemicarbazone N_2_S ligand as one might expect occupies the pentagonal plane. This Bi(III) complex nonetheless was found to be significantly less active (IC_50_ = 46.2 µM) than cisplatin (1.2 µM) and indeed the free ligand (12.3 µM) against K562 (leukaemia) cells on 24 h treatment [92].

A separate report later in 2012 features a Bi(III) complex of 2-acetylpyridine, *N*(4)-pyridyl thiosemicarbazone (HL), [Bi(HL)(NO_3_)_3_] [91]. The molecular geometry of this 9-coordinate complex, accounting for the inert electron pair (6s^2^), is one dodecahedral. The neutral thiosemicarbazone coordinates the Bi(III) via its pyridine nitrogen, imine nitrogen and thione sulphur to give two five-membered chelate rings, Figure 16b. This complex exhibits good cytotoxicity (IC_50_ <10 µM) against a panel of cancer cell lines; K562, (leukaemia), HCT116 (colorectal), HepG2 (hepatocellular) and HeLa (cervical) cancer cells. Significantly and in contrast to the 2-acetylpyrazine *N*(4)-phenylthiosemicarbazone complex exhibits similar cytotoxicity (1.8 µM) as cisplatin (1.2 µM) and superior cytotoxicity than the free ligand (>100 µM) and Bi(NO_3_)_3_ (41.2 µM) against the K562 leukaemia cells. Results from annexin-V FITC/PI double staining and caspase-3 activation experiments on HepG2 cells treated with this complex, suggest that the cytotoxicity observed may be due to induction of apoptosis [91].

**Figure 17:**
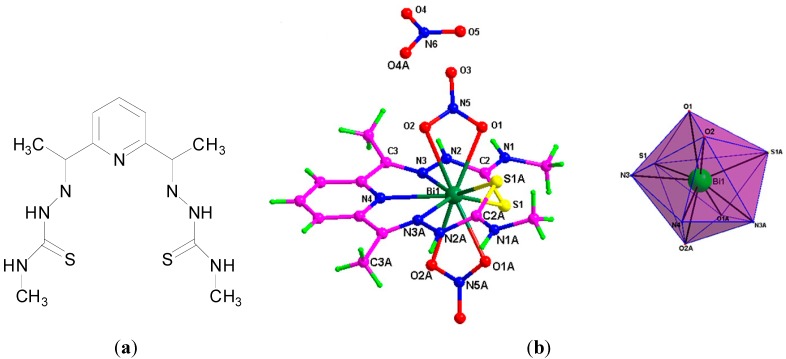
(**a**) Structure of 2,6-diacetylpyridine bis(^4^*N*-methylthiosemicarbazone) (H_2_L); (**b**) Molecular structure of [Bi(H_2_L)(NO_3_)_2_]NO_3_ atomic numbering and polyhedral showing distorted geometry around the Bi atom. Reprinted with permission from [19]. Copyright (2012) American Chemical Society.

A Bi(III) complex of the pentadentate ligand 2,6-diacetylpyridine bis(^4^*N*-methylthiosemicarbazone), H_2_L, Figure 17a, [Bi(H_2_L)(NO_3_)_2_]NO_3_ was also reported [19]. The Bi(III) centre is nine-coordinate where the neutral pentadentate thiosemicarbazone ligand coordinates via three nitrogens and two sulphur atoms (N_3_S_2_) and two nitrate ions coordinate via the four oxygen atoms as shown in the X-ray crystal structure (Figure 17b).

The *in vitro* cytotoxicity of this complex was investigated also against the K562 leukaemia cancer cell line for 24 h. The Bi(III) complex has a lower *in vitro* IC_50_ value (26.8 µM) than both the free ligand (H_2_L, 82.3 µM) and Bi(NO_3_)_3_·5H_2_O (41.2 µM) but not compared to the previously reported 2-acetylpyridine, *N*(4)-pyridylthiosemicarbazone complex (Figure 16b) which has an IC_50_ value of 1.8 µM. Regardless the activity of this complex was investigated *in vivo* in Kunming mice by caudal injection [19]. The LD_50_ was determined to be 44.7 mg/kg and effective H22 xenograft tumour growth inhibition (61.6%) was demonstrated after treatment at 10 mg/kg [19].

**Figure 18:**
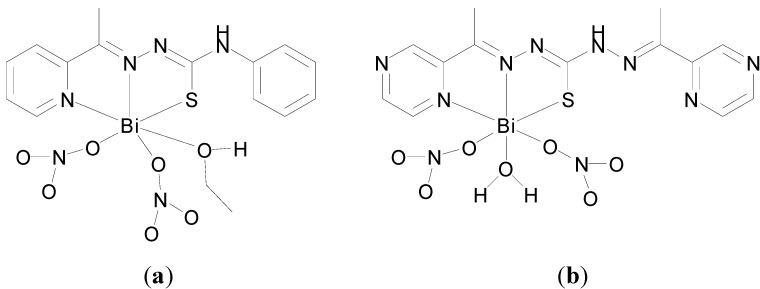
Structure of (**a**) a Bi(III) 2-acetylpyridine *N*(4)-phenylthiosemicarbazone (HL) complex, [Bi(L)(NO_3_)_2_(CH_3_CH_2_OH)]; (**b**) a Bi(III) bis(2-acetylpyrazine) thiocarbonohydrazone (H_2_L) complex, [Bi(HL)(NO_3_)_2_(H_2_O)].

Yan-Ke Li *et al.* developed a 7-coordinate Bi(III) thiosemicarbazone complex but of 2-acetyl- pyridine-*N*(4)-phenylthiosemicarbazone (HL), [Bi(L)(NO_3_)_2_(CH_3_CH_2_OH)] (Figure 18a). This complex differs only by the substitution of a pyridine ring for a pyrazine ring and a solvating ethoxide as opposed to a methoxide in the 2-acetylpyrazine *N*(4)-phenylthiosemicarbazone complex, Figure 16a, though displays far superior cytotoxicity activity [93]. It has sub-10 µM IC_50_ values against K562, HCT116, HepG2 and HeLa cancer cells and somewhat similar activity as cisplatin against K562 cells as was the case for 2-acetylpyrazine *N*(4)-phenylthiosemicarbazone complex, Figure 16b. Li *et al.* provide a valuable insight into the potential mechansim of action of this class of compounds by demonstrating that 2-acetylpyridine-*N*(4)-phenylthiosemicarbazone Bi(III) complex promotes a dose-dependent apoptosis in HepG2 cells, which is associated with an increase in intracellular concentrations of ROS and reduction in mitochondrial membrane potential [93].

There has been considerable interest in the development of Bi(III) thiosemicarbazone complexes as both potential anti-cancer and anti-bacterial agents, which has resulted in the development of interesting complexes with some good activity. Nonetheless the strategy of coordinating Bi(III) to thiosemicarbazones to enhance the activity of the ligands clearly does not always work [92]. Therefore the development of effective Bi(III) complexes of thiosemicarbazones as anti-cancer agents will certainly require a greater understanding of the activity of the ligands and biomolecular effects of the corresponding Bi(III) complexes.

Zhang *et al.* developed Bi(III), Ga(III) and diorgano Sn(IV) complexes of bis(2-acetyl- pyrazine)thiocarbonohydrazone (H_2_L). The Bi(III) complex, [Bi(HL)(NO_3_)_2_(H_2_O)] (Figure 18b) was found to be the most cytotoxic of the compounds studied and 14-fold more cytotoxic than the free thiocarbonohydrazone ligand against HepG2 liver cancer cells [94]. The authors preliminary investigation into a possible mechanism of action of these complexes suggest that the apoptosis observed is connected with generation of reactive oxygen species (ROS) production and also reduction of mitochondrial membrane potential [94].

**Figure 19:**
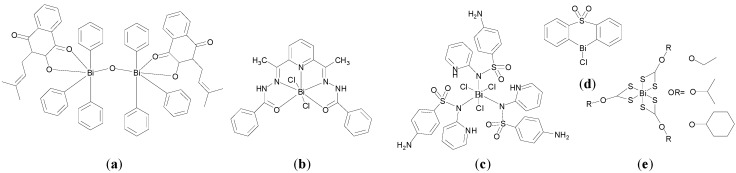
Structure of (**a**) (Lp)_2_(Ph_3_Bi)_2_O, (**b**) [Bi(HAcPh)Cl_2_], (**c**) [BiCl_3_(Spy)_3_], (**d**) bichlorodibenzo[c,f][1,5]thiabismocine compound and (**e**) general structure of Bi xanthate complexes.

Three recent publications from Belo Horizonte, Brazil describe novel Bi(III) complexes with cytotoxic activities. De Oliveira and co-workers developed a Bi(V) complex of lapachol (Lp), a natural product with reported anti-tumour, anti-malarial and anti-leshmanial properties. Such properties are attributed to reactive oxygen species generation and/or DNA interchelation [117]. The dinuclear Bi(V) complex, (Lp)_2_(Ph_3_Bi)_2_O, where the Bi atoms are bridged by one O atom, Figure 19a, was found to have a favourable IC_50_ value (1.8 µM) as compared to cisplatin (1.0 µM) against K562 cells. Significantly this Bi(V) complex was also more cytotoxic than the free ligand (*ca*. 5-fold), a reported Sb(V) lapochol complex, (Lp)(Ph_3_Sb)OH (c. 20-fold) and Ph_3_BiCl_2_ (c. 17-fold) and determined to be stable in aqueous solution [117]. This is a rare example of an organobismuth(V) complex that exhibits noteworthy *in vitro* cytotoxicity.

Ferraz *et al.* report a series of Bi(III) complexes of 2,6-diacetylpyridine bis(benzoylhydrazone) derivatives. Hydrazone derivatives are known to possess anti-microbial and anti-tubercular activities [95]. The Bi(III) hydrazone complex, [Bi(HAcPh)Cl_2_] (Figure 19b) where H_2_AcPh is 2,6-diacetylpyridine bis(benzoylhydrazone), proved to be most active against a panel of cancer cell lines (Jurkat, HL60, MCF-7 and HCT-116). This compound was more cytotoxic than the free ligand, corresponding Sb(III) complex, [Sb(HAcPh)Cl_2_] and also cisplatin. The reported Bi(III) complex of the corresponding *p*-choro-benzoylhydrazone derivative, [Bi(HAcpClPh)Cl_2_], perhaps represented the best drug candidate though as it possessed a superior therapeutic index once activities against the normal cell line, PBMC, were considered [95].

Subsequently Marzano and co-workers developed a novel bismuth complex of sulfapyridine (SpyH), [BiCl_3_(Spy)_3_] (Figure 19c) [118]. Sulfapyridine is a sulfonamide anti-bacterial, the first synthetic antibiotics to be administered clinically. It is no longer used medicinally in humans in part due to resistance but also a potential risk of developing agranulocytosis or leukopenia. Nonetheless the Bi(III) complex of sulfapyridine was demonstrated to inhibit the growth of leukemia cells, K562, with an IC_50_ of 44 µM at 72 h treatment. Sulfapyridine in contrast had an IC_50_ > 100 µM against the same cell line [118].

In Japan, Iuchi *et al.* investigated the anti-cancer activities of a family of heterocyclic organobismuth(III) compounds previously investigated as an anti-microbial agents [100,119]. These compounds exhibited significant anti-cancer activity against a broad panel of cancer cell lines [120]. The authors focused on the activity of the bi-chlorodibenzo[c,f] [1,5]. thiabismocine compound (Figure 19d) against the human promyelocytic leukemia cell line, HL-60, due to (i) its submicromolar IC_50_ values against a panel of leukemia cell lines at 12 h treatment (e.g., HL-60, IC_50_ = 0.151 µM) and (ii) the propensity of the fellow group 5 compound arsenic trioxide to effectively treat acute promyelocytic leukemia [3]. It is noteworthy that the authors undertook a relatively comprehensive investigation into the mechanism of action of this heterocyclic organobismuth(III) compound.

The compound induced apoptosis at low concentrations (0.22–0.44 µM) resulting in internucleosomal DNA degradation, nucleus condensation/fragmentation and laddering [120]. At higher concentrations (>1.1 µM) acute necrosis was observed. Intracellular ATP levels and/or ROS concentration were proposed as key factors determining cell death by apoptosis or necrosis. Caspase-3 (effector) and caspase-9 (initiator, intrinsic or mitochondrial pathway) were determined to participate in the observed apoptosis whereas caspase-8 (initiator, extrinsic or death receptor signaling pathway) to a lesser extent. Iuchi *et al.* surmised that generation of ROS and release of cytochrome from mitochondria contributed to the anti-cancer activity of these compounds.

An interesting article authored by Friebolin *et al.* describes the anti-cancer activity of a series of xanthate complexes of Bi, Cu, Au, Ni, Pd and Rh. Eighteen bis(O-alkyldithiocarbanato) metal complexes were synthesized in total and their anti-cancer activity at pH 6.8 and 7.4 against Calu-6 (lung) and MCF-7 (breast) cancer cell lines were investigated and compared to the activity of cisplatin and bis(O-alkyldithiocarbanato) Pt complexes. The Pd complexes performed the best, followed by Bi complexes such as those shown in Figure 19e. Bi(S_2_COR)_3_ where R is isopropyl for example has an IC_50_ of 11.2 µM as compared to cisplatin’s 27.21 µM. In addition the Bi complexes were also found to be more active at pH 6.8 than 7.4 [121]. pH 6.8 was investigated given the assertion that solid tumours often have pH’s of 6.8 and lower [121].

**Figure 20:**
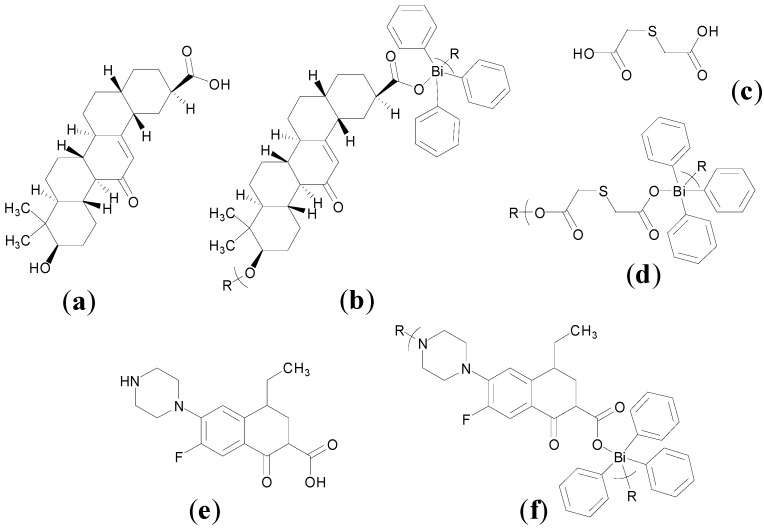
Structures of (**a**) GA, (**c**) TG and (**e**) NF and proposed structures for repeat units of the polymers; (**b**) BiPh_3_/GA, (**d**) BiPh_3_/TG and (**f**) BiPh_3_/NF where R is a chain extension.

Carraher, Jr. and coworkers are interested in the development of biomedically or biologically active Group VA metal-containing polymers [122]. Recent example of this work describe organoarsenic, oraganoantimony and oragnobismuth polymers of biologically active monomers such as glycyrrhetinic acid (GAH_2_) [123], thiodiglycolic acid (TGH_2_) [124] and norfloxacin (NFH_2_) [125] as potential anti-cancer agents, Figure 20. IR spectroscopy, ^1^H-NMR and MALDI MS were employed to characterise the reported polymers.

GA for example is a pentacyclic triterpenoid with medicinal properties. It is an inhibitor of 15-hydroxyprostaglandin dehydrogenase and delta-13-prostaglandin which gives rise to increased levels of prostaglandins. It is also is a potent inducer of mitochondrial permeability transition and can therefore trigger the pro-apoptotic pathway [123]. The organometallic Bi polymer of GA, BiPh_3_/GA, is described as a mixture of bridging and non-bridging structures about the Bi though largely non-bridging and asymmetric. Characterisation is consistent with the formation of poly ether esters, Figure 20b. The reported chain length is 2500. The organometallic Bi polymer of GA, BiPh_3_/GA exhibited moderate activity against a pancreatic cell line (PANC-1, EC_50_ = 3800 ng/mL) though cisplatin (AsPC-1, EC_50_ = 1400 and PANC-1, EC_50_ = 340 ng/mL) and the organometallic antimony polymer of GA, SbPh_3_/GA (AsPC-1, EC_50_ = 1900 and PANC-1, EC_50_ = 1700 ng/mL) exhibited superior activity against both pancreatic cell lines tested [123].

The polyamine ester organometallic Bi polymer of norfloxacin, BiPh_3_/NF (Figure 20f) represents a better example of the anti-cancer potential of Bi containing polymers. Norfloxacin as previously mentioned is a fluoroquinolone broad spectrum antibiotic. The BiPh_3_/NF polymer is predominantly a bridging structure about the Bi and IR spectroscopy indicates the formation of Bi-O and Bi-N bonds. The reported chain length is 2100. The Bi polymer exhibited superior activity than the antimony and arsenic-base polymers in four (pancreatic, breast, colon and prostate) out of the six cancer cell lines tested with marked activity against the pancreatic cell line, PANC-1 (EC_50_ = 14 µg/mL) which was also superior to that of cisplatin (EC_50_ = 340 µg/mL). The Bi polymer also exhibits selectivity when its cytotoxicity for the breast cancer cell line, MDA MB-231 (EC_50_ = 0.61 µg/mL) is compared to its toxicity towards the WI-38 normal embryonic human lung fibroblast cells (EC_50_ = 4.2 µg/mL). The results in this study are encouraging and the anti-cancer activity of Bi containing polymers certainly merits further investigation.

Many of the Bi-containing compounds developed to date and evaluated as potential anti-cancer agents were predominantly developed: (i) given that Bi-containing compounds have exhibited cytotoxicity in the past or exhibit other “biological activity” such as anti-bacterial activity or (ii) as Bi-containing complexes which possess ligands previously determined to be cytotoxic. Tentative references have been made to the presence of Bi potentially enhancing the lipophilicity of particular ligands or indeed ligands and Bi acting in a synergistic manner. It is also noteworthy that such compounds appear to exhibit more favourable cytotoxicity against leukaemias as opposed to cancers associated with solid tumours, which resonates with the activity of As_2_O_3_ for example.

In the main there is a distinct lack of information regarding potential mechanisms of action of Bi-containing compounds as anti-cancer agents though recently investigators have strived to gain a better understanding in relation to how their compounds may exert their cytotoxic effects. Presumably Bi(III) targets non DNA sites and in turn Bi-containing compounds possess different anti-cancer mechanisms of action as compared to classical Pt anti-cancer drugs. The recent elucidation of PML, a zinc finger protein, as being the target for As_2_O_3_ in relation to its activity against APL and provision of evidence that As_2_O_3_ interacts with cysteines located in zinc fingers within the RING domain and B2 motif of PML, may serve as inspiration for those interested in discovering a relevant Bi(III) anti-cancer target [3].

The growing interest in the development of novel Bi-containing compounds as anti-cancer agents is extremely positive and the activity of reported compounds certainly supports the view that Bi-containing drugs do have a potential role to play as effective anti-cancer agents. Nonetheless a more rational approach to the design of such compounds is desirable and surely will be more productive. Identification of the exact role Bi in targeting cancer is a prerequisite. Subsequently the challenge of delivering Bi to the target must be met whether it by selection of appropriate ligands for the development of Bi complexes or design of bespoke organobismuth compounds.

Radiometals such as ^212^Bi and ^213^Bi are also used to develop therapeutic radiopharmaceuticals. Radiotherapy is the use of high energy radiation to kill cancer cells and shrink tumours. ^212^Bi and ^213^Bi are α-emitting radionuclides with half-lives of 60.6 and 45.6 min respectively [126]. α-Emitting ^212^Bi and ^213^Bi containing radiopharmaceuticals should be ideally suited to treat cancer as the short particle range and high energy deposition can be used to kill cancer cells by damaging DNA directly after administration of a dose at a tumour site [127]. Such radiation could be used to target tumours defective in double strand breaks repair mechanisms for example. In addition they decay ultimately to stable isotopes and offer less radiotoxicity, as compared to β^−^-emitters for example, due to their shorter particle range. ^213^Bi is thought to be best suited though, as one of the daughter radionuclides associated with ^212^Bi is ^208^Tl, which emits a 2.6 MeV gamma ray.

A number of targeted ^212^Bi and ^213^Bi-based therapeutic radiopharmaceuticals have been developed and even assessed in preclinical and clinical trials involving cancer patients with leukemia, melanoma, and glioblastoma [127,128,129,130,131,132]. Ideally radiopharmaceuticals should have high tumour uptake, a high tumour to background ratio, long residence time and fast renal clearance [127]. Targeted radiopharmaceuticals generally consist of a bifunctional chelate (BFC), which simultaneously binds a radiometal and often via a linker, a targeting biomolecule such as a peptide or monoclonal antibody [3].

**Figure 21:**
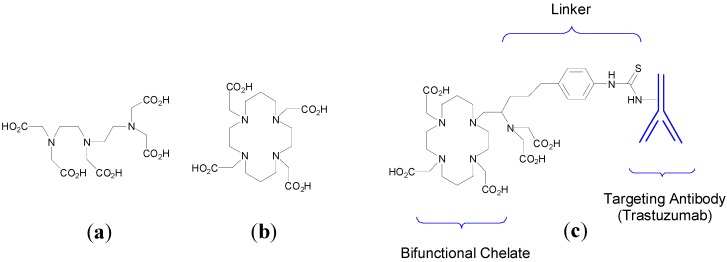
Structures of (**a**) DTPA and (**b**) DOTA, which are representative examples of common chelating agents for ^212^Bi and ^213^B. (**c**) Representation of 3p-C-DEPA conjugated to trastuzumab.

Typical chelating agents, which are functionalised, include for instance diethylenetriamine pentaacetic acid, DTPA, Figure 21a, and 1,4,7,10-tetraazacyclododecane-1,4,7,10-tetraacetic acid, DOTA, Figure 21b. Song *et al.* developed for example a novel bifunctional chelate ligand, 3p-C-DEPA, for use in targeted α-radioimmunotherapy (RIT) of ^212^Bi and ^213^Bi. The bifunctional ligand was conjugated with trastuzumab, Figure 21c, a human epidermal growth factor receptor 2 (HER2) targeting antibody, which selectively targets the HER2 protein overexpressed in particular cancers including 90% of colorectal cancers [126].

Cherel and co-workers recently investigated the potential of ^213^Bi radioimmunotherapy in a murine model of multiple myeloma [131]. Specifically they wished to establish efficacy in multiple myeloma minimal residual disease treatment in mice with low tumour burden. An antimouse CD138 antibody was radiolabelled with ^213^Bi where the antibody was modified with the 2-(4-isothiocyanatobenzyl)-cyclohexyl-DTPA chelator. CD138 antigen was targeted as it is strongly expressed on myeloma cells in all patients. RIT at 3.7 and 7.4 MBq exhibited a median survival greater than 303 and 227 days respectively as compared to the untreated control groups 45.5 days. Furthermore only moderate and transient toxicity was associated with treatment [131].

Wild *et al.* reported on the efficacy of two α-emitting ^213^Bi-labelled peptides, ^213^Bi-DOTA-PESIN and ^213^Bi-AMBA in a human prostate cancer xenograft model, where PESIN is PEG_4_-bombesin and AMBA is DO3A-CH_2_CO-8-aminooctanoyl-Q-W-A-V-G-H-L-M-NH_2_) for peptide-receptor radio-nuclide therapy (PRRT). It is noteworthy that potential advantages of PRRT using small peptides over RIT is lack of immunogenicity, fast diffusion and fast blood clearance, which can complement ^213^Bi half-life of 45.6 min [129]. The peptides are analogues of gastrin releasing peptide (GRP) and target the GRP receptor, which is expressed on all invasive prostatic carcinomas but not benign prostatic tissue at high density. Significantly at MTD’s (25 MBq) both ^213^Bi-labelled peptides were more effective than the ^177^Lu-DOTA-PESIN where ^177^Lu is a β^−^-emitting isotope, indicating that the α-therapy was found to be better than β^−^-therapy in this study.

RIT and PRRT with ^213^Bi-labelled antibodies or peptides respectively have the potential to be safe and effective therapeutic approaches. We await the results of ongoing clinical trials with interest.

Bi may also have a number of indirect roles to play in relation to treating cancer/tackling the incidence of cancer. Bi has been linked with the alleviation of side effects associated with cisplatin treatment for example [133,134,135]. Nephrotoxicity is a significant dose-limiting factor in cisplatin-based chemotherapy. Administration of Bi(III) salts prior to cisplatin treatment has been shown to depress the renal toxicity associated with cisplatin treatment. This renoprotective effect is thought to be due to Bi increasing metallothionein production in the kidneys [24,133,134,135]. More recently, Leussink *et al.* investigated the genomic basis of the renoprotective effect by treating NRK-52E cells, a cell line of rat proximal tubular epithelial origin, with Bi(III) salts. Subtraction PCR and microarray techniques were used to detect differentially expressed genes in treated and untreated NRK-52E cells. Down regulated (0.17–0.31-times) genes include cytochrome c oxidase subunit I, BAR (an apoptosis regulator), heat-shock protein 70-like protein, and ribosomal proteins S7 and L17, and S1, proteins belonging to the translation machinery. The single gene to be identified as being upregulated was glutathione S-transferase subunit 3A [136].

In addition and as referenced in Section 2, the link between *H. pylori* and gastric cancer, the second most common cause of cancer death in the world, is well established [48,137]. *H. pylori*-induced chronic gastritis can be the first step in the so-called multistep cascade of gastric cancer [138]. In addition and more recently *H. pylori* has been linked with extragastric diseases including gallbladder cancer [49]. Therefore the development of effective Bi-based treatments for *H. pylori* could in turn indirectly have positive effects on the incidence of both gastric and gall bladder cancer worldwide.

## 6. Conclusions

Bismuth-based drugs are routinely used for the treatment of gastrointestinal disorders and are found as key components of quadruple therapeutic regimens which are becoming increasingly common as first-line treatment options for *H. pylori*. Bismuth also has significant anti-microbial, anti-leishmanial and anti-cancer properties. In turn much research has been undertaken recently in relation to the synthesis, characterisation and evaluation of novel Bi-based compounds as potential anti-microbial, anti-leishmanial and anti-cancer agents. Significantly many of these compounds exhibit excellent activity. It is likely that proteins are key biomolecular targets of Bi, particularly proteins which possess cysteine (thiol) rich domains or metal binding sites with attractive coordination environments for Bi.

Metallomic/metalloproteomic approaches will certainly help further elucidate the: (i) mechanism of action of Bi on biomolecules; (ii) mechanisms of resistance employed by microbial, protozoan and cancer cells and (iii) mechanisms of biological uptake, storage and removal of Bi [139].

^212^Bi and ^213^Bi containing radiopharmaceuticals also hold much promise as effective α-emitting anti-cancer agents. Radiolabelling suitable bifunctional chelating ligands with ^212^Bi and ^213^Bi and selection of effective targeting biomolecules is the strategy of choice.

Given the notable resurgence of interest in the bioinorganic chemistry of Bi and development of novel classes of Bi compounds with considerable and varied biological activity, it is becoming increasingly apparent that Bi does have the potential to play new and important roles in medicinal chemistry.
